# Probing Excited-State Electronic Structure and Dynamics of Fluorescein Doped in PVA Film Using Electroabsorption and Electrophotoluminescence Spectroscopy

**DOI:** 10.1021/acs.jpcb.6c02869

**Published:** 2026-07-27

**Authors:** Shailesh Rana, Rong-Lin Jhang, Kamlesh Awasthi, Nobuhiro Ohta

**Affiliations:** † Department of Applied Chemistry and Institute of Molecular Science, 34914 National Yang Ming Chiao Tung University, 1001 Ta-Hsueh Road, Hsinchu 300093, Taiwan; ‡ Centre of Energy Research, Graphic Era Deemed to be University, Bell Road, Clement Town, Dehradun 248002, India; § Center for Emergent Functional Matter Science, National Yang Ming Chiao Tung University, 1001 Ta-Hsueh Road, Hsinchu 300093, Taiwan

## Abstract

Electroabsorption (E-A) and electrophotoluminescence (E-PL) spectra of fluorescein (FL) doped in poly­(vinyl alcohol) (PVA) film were measured in the visible region at various concentrations of FL in PVA. By analyzing the E-A spectra, the changes in the electric dipole moment and polarizability following excitation into the S_1_ state are determined for each concentration. The analysis of the E-A spectra indicates the formation of H-aggregates of FL in PVA at high concentrations. The electric-field-induced changes in the radiative decay rate constant (*k*_r_) of FL in PVA, estimated from the field-induced change in the total absorption intensity, indicate that *k*_r_ decreases in the presence of an electric field and that the magnitude of the field-induced change in *k*_r_ decreases with increasing FL concentration. In contrast to the *k*_r_ of the FL monomer, the *k*_r_ of H-aggregates is considered to increase with the application of an electric field. The fluorescence lifetime of FL in the PVA film decreases rapidly with increasing FL concentration, indicating a monotonic decrease in the fluorescence quantum yield with increasing FL concentration due to the rapid increase of the rate constant of the nonradiative decay process (*k*_nr_). The nonradiative process from the emitting state of FL is probably assigned to the energy dissipation to nonemissive H-aggregates, i.e., energy transfer from the emitting state of the FL monomer to the H-aggregate. The applied electric field quenches the fluorescence of FL in PVA, indicating the field-induced increase in *k*_nr_. Further, the magnitude of the field-induced quenching increases rapidly with increasing FL concentration, indicating that the field-induced increase of *k*_nr_ increases significantly with increasing FL concentration in PVA.

## Graphical abstract

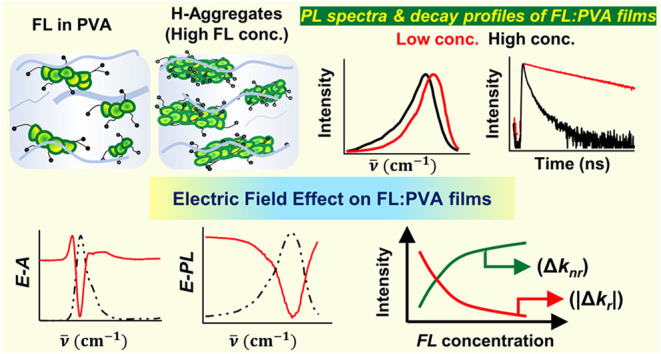

## Introduction

Organic fluorescent dyes have long played an important role in the development of optoelectronic materials due to their strong light–matter interactions, tunable electronic structures, and solution processability.^1–10^ Among these, fluorescein (FL) stands out as a prototypical xanthene-based dye that is the most widely studied and utilized organic fluorophore, and is renowned for its intense visible absorption and fluorescence spectra, high emission quantum yield, and versatile chemical functionality.^11^ FL has played a pivotal role in the development of fluorescence-based techniques in various disciplines, including chemistry, physics, biology, medicine, and materials science.^12–15^ These make FLs not only a benchmark fluorophore in spectroscopy but also an attractive model system for investigating fundamental photophysical studies and a practical probe in applied research.

FL possesses a rigid conjugated xanthene core that is responsible for strong π–π* electronic transitions in the visible region under alkaline or weakly polar conditions.^16^ The presence of phenolic and carboxylic functional groups introduces rich acid–base chemistry. In aqueous solutions, it exists in cationic, neutral, anionic, and dianionic forms, making its absorption and fluorescence properties strongly pH-dependent.^17,18^ These different species exhibit distinct absorption and emission spectra, making fluorescein a classic pH indicator and benchmark system for studying proton-coupled photophysics. This strong dependence on the chemical environment makes fluorescein highly sensitive to local polarity, hydrogen bonding, and electric fields.

In the field of optoelectronics, FL has attracted considerable attention as an active medium operating in the visible spectral range,^4,6–9^ owing to its favorable fluorescence efficiency and adequate photostability. High-quality thin films of FL have been successfully fabricated using solution-based techniques, such as spin coating, and their optical characteristics indicate a strong potential for integration into optoelectronic and nonlinear optical devices.^7–9^ Beyond light-emitting applications, fluorescein has been extensively employed in sensor technology. For instance, photonic crystal fibers infiltrated with aqueous fluorescein solutions have been demonstrated as multifunctional sensors capable of simultaneously detecting ultraviolet radiation and temperature variations.^12^ In the solid state, fluorescein-based luminescence platforms have been developed for relative humidity sensing and show promise for vapor detection applications.^19^ Furthermore, chemically modified fluorescein derivatives have been reported as highly sensitive ON–OFF fluorescent chemosensors, particularly for applications involving trace-level blood detection.^20^ In the solid state or when embedded in polymer films, further, fluorescein serves as an excellent model system for studying intermolecular interactions, aggregation effects, and environment-induced changes in optical properties.^21–23^

Molecular aggregation strongly influences the photophysical behavior of organic chromophores in condensed phases. As the concentration of fluorescent dye molecules increases, aggregation becomes more pronounced, affecting properties that are important for applications such as nonlinear optics, solar cells, and optical memory devices. However, the impact of the electric field on the electronic structure and dynamics in the photoexcited state with molecular aggregation, that is, field-induced changes in the electric dipole moment and polarizability, and field-induced changes in the radiative and nonradiative relaxation processes, is less understood. It is strongly expected that investigating the concentration dependence of electroabsorption (E-A) and electrophotoluminescence (E-PL) spectra of fluorescein doped in a polymer film, i.e., field-induced change in the absorption and photoluminescence (PL) spectra of fluorescein, provides an opportunity for understanding the relationship between aggregation and electric-field-induced change in the electronic structure and dynamics in the excited state.

In the present study, we measured the electroabsorption (E-A) and electrophotoluminescence (E-PL) spectra, i.e., field-induced changes in the absorption and photoluminescence (PL) spectra of FL doped in poly­(vinyl alcohol) (PVA) film at different FL concentrations. The analysis of the E-A spectra enables the quantitative evaluation of photoinduced changes in the electric dipole moment and molecular polarizability, as well as the field-induced change in the radiative decay rate constant (*k*_r_) in the excited state. Furthermore, the field-induced modulation of relaxation dynamics is examined through the measurements of E-PL spectra, with which the field-induced change in the nonradiative decay rate constant (*k*_nr_) can be estimated in combination with the PL lifetime. These results provide insights into the mechanisms underlying the electric-field-induced changes in the photoexcitation dynamics of FL doped in PVA film. Since the emission quantum yield of FL is usually very high, it may also be expected that the present results are applicable for estimation of the strength of the local electric field in molecular systems and environments, e.g., by attaching an FL molecule to DNA or doping FL in materials.

## Experimental Methods

Fluorescein (99%), poly­(vinyl alcohol) (PVA, 99%), and sodium hydroxide (NaOH, 99%) were obtained from Sigma-Aldrich and used without further purification. PVA was dissolved in deionized water (0.16 g in 4 mL) at 80 °C under stirring for 4–5 h. Fluorescein was separately dissolved in 0.1 M NaOH (0.3 mL) to prepare dye concentrations of 0.4, 1.0, 2.0, 3.0, 4.0, and 4.6 wt %. These solutions were filtered and mixed prior to film deposition. Cleaned SiO_2_ substrates were prepared by detergent washing, ultrasonication in acetone/isopropanol/deionized (DI) water (1:1:1), and oven drying at 100 °C. Fluorescein:PVA thin films were deposited by spin coating (420 rpm for 60 s, followed by 1000 rpm for 10 s), yielding a thickness of ∼700 nm. A 40 nm Al top electrode was thermally evaporated to form devices with a quartz/indium tin oxide (ITO)/SiO_2_/fluorescein:PVA/Al architecture.

UV–vis absorption and PL spectra were recorded using JASCO V-780 and FP6300 spectrometers, respectively, with an excitation wavelength of 470 nm for PL. E-A and E-PL spectra were recorded using a spectrometer (JASCO-FP-777 and FP6300).^24,25^ Briefly, a sinusoidal alternating current (AC) voltage (frequency of 40 Hz) was generated by a function generator (SG-4311, Iwatsu) and further amplified by a high-voltage amplifier. This voltage was then applied between the ITO and Al electrodes of the sample, thereby creating an alternating electric field across the device. Field-induced changes in the transmitted excitation light intensity and PL intensity were detected using a lock-in amplifier (SR830, SRS) at the second harmonic of the modulation frequency of the applied electric field. For the E-PL measurements, the excitation wavelength was chosen to minimize the field-induced absorption effects.

Time-resolved PL decay measurements were carried out using an inverted microscope (TE2000E, Nikon), integrated with a Becker & Hickl time-correlated single-photon counting system (TCSPC) (GmbH, SPC-830) and a Ti-Sapphire laser (Tsunami, Spectra-Physcis; a pulse width of 80 fs and a repetition rate of 81 MHz) as an excitation light source. The excitation laser light was tuned to 900 nm and frequency-doubled to 450 nm using an ultrafast harmonic system (Model 5-050, Inrad).^24^ The excitation power at the fiber tip was maintained at ∼1 mW by employing a neutral density filter. Emission decay from the samples was collected by a photomultiplier tube (R3809U, Hamamatsu).

## Results and Discussion

### Absorption and Photoluminescence (PL) Characteristics of Fluorescein (FL)

The UV–visible absorption and PL spectra of FL doped in PVA film, hereafter denoted as FL:PVA film, were observed for FL concentrations ranging from 0.4 to 4.6 wt %, as shown in Figure 1. The absorption spectra exhibit a maximum at ∼505 nm at all concentrations, with a shoulder at ∼480 nm due to the vibrational nature. The PL spectra of FL:PVA film show a red shift with increasing concentration, e.g., the peak positions are 522, 526, and 531 nm at 0.4, 2.0, and 4.0 wt %, respectively. Both the absorption and PL spectra of FL:PVA film show a red shift compared with the spectra in solution, but the spectral shapes are similar. The absorption and PL spectra of FL in solution, shown in Supporting Information (SI, Figure S1), have peaks at 490 and 512 nm, respectively. The red shift of the absorption and PL spectra of FL:PVA film, compared to the dye in solution, is attributed to the modification of the electronic states of the FL molecules at the polymer–dye interface.^8^ In addition to the FL molecules that show slightly red-shifted PL, aggregates of FL that operate as acceptors for excitation energy transfer from the fluorescent PL may be generated with increasing FL concentration in PVA film. The presence of an efficient energy transfer process from the PL state is supported by the concomitant decrease in the PL lifetime with increasing FL concentration from 0.4 to 4.6 wt %. The observed PL decay profiles show a monotonic increase in the decay rate with increasing FL concentration, as shown in Figure 2. The average lifetimes (τ) were 2.51, 0.91, 0.40, 0.30, 0.17, and 0.16 ns at FL concentrations of 0.4, 1.0, 2.0, 3.0, 4.0, and 4.6 wt %, respectively (see Table 1), indicating that the lifetime monotonically and rapidly decreased with increasing FL concentration. The τ is given by 1/(*k*_r_ + *k*_nr_), where *k*_r_ and *k*_nr_ are the radiative and nonradiative decay rate constants, respectively, and the quantum yield of PL (Φ_PL_) is given by *k*_r_/(*k*_r_ + *k*_nr_). The *k*_r_ value of fluorescein was estimated by using the Strickler–Berg relation^26^ to be 2.37 × 10^8^ s^–1^ in a diluted FL solution (1 μM) with the absorption and steady-state PL spectra shown in Figure S1. The peak positions in the absorption and PL spectra of FL in the PVA film are slightly different from those in solution, but the spectral shapes are very similar (cf. Figures 1 and S1), and thus the *k*_r_ value is considered to be the same in both the solution and PVA films. Then, Φ_PL_ was estimated by using the relation Φ_PL_ = *k*_r_τ. The *k*_nr_ of FL in FL:PVA film was also estimated using the relation *k*_nr_ = 1/τ – *k*_r_. The obtained Φ_PL_ and *k*_nr_ are shown in Table 1 and Figure 3. Φ_PL_ and *k*_nr_ monotonically and rapidly decrease and increase, respectively, with increasing FL concentration in FL:PVA film, which is related to the monotonic and rapid shortening of τ. The PL lifetime shortening with increasing FL concentration reflects the enhanced nonradiative relaxation pathways associated with the reduced intermolecular distance. As mentioned later, the formation of H-aggregates of FL in PVA film with increasing concentration is responsible for the rapid decrease in the fluorescence lifetime, caused by efficient energy transfer from the emitting state of PL to aggregates.

**Figure 1:**
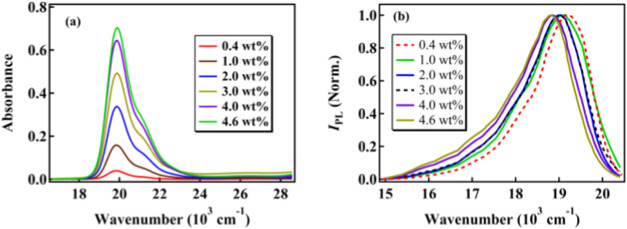
UV–vis absorption (a) and PL (b) spectra of FL doped in PVA film at different concentrations. The excitation wavelength used for PL measurements was 470 nm in all cases.

**Figure 2:**
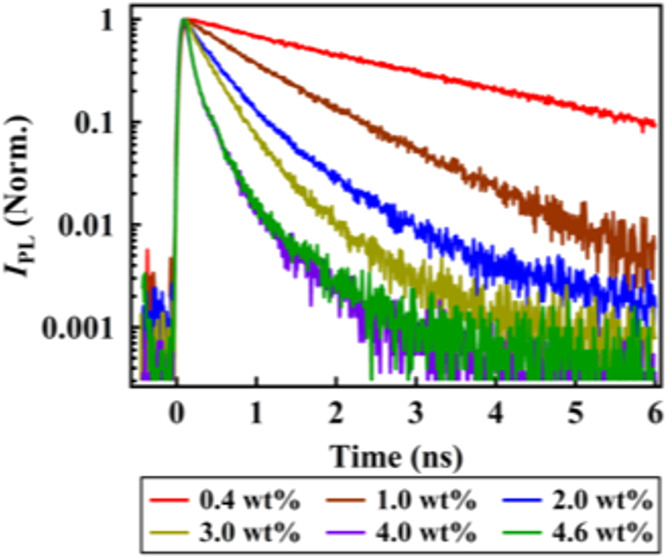
PL decay profiles of FL:PVA film with different FL concentrations. The excitation wavelength was 450 nm in all cases.

**Table 1:** Fluorescence Lifetime (τ), Fluorescence Quantum Yield (Φ_PL_), Nonradiative Decay Rate (*k*_nr_), Field-Induced Change in the Radiative Decay Rate (Δ*k*_r_), Field-Induced Change in the PL Intensity Relative to the PL Intensity at Zero Field (Δ*I*_PL_/*I*_PL_), and Field-Induced Change in the Nonradiative Decay Rate (Δ*k*_nr_) at Different FL Concentrations^a^

concentration (wt %)	τ (ns)	Φ_PL_	*k*_nr_ (10^9^ s^–1^)	Δ*k*_r_ (10^4^ s^–1^)	Δ*I*_PL_/*I*_PL_ (%)	Δ*k*_nr_ (s^–1^)
0.4	2.51	0.59	0.16	–4.74	–0.02	4.7 × 10^4^
1.0	0.91	0.22	0.86	–1.72	–0.14	1.4 × 10^6^
2.0	0.40	0.09	2.26	–0.89	–0.18	4.1 × 10^6^
3.0	0.30	0.07	3.09	–0.93	–0.20	5.7 × 10^6^
4.0	0.17	0.04	5.65	–1.17	–0.37	2.3 × 10^7^
4.6	0.16	0.04	6.01	–1.02	–0.30	1.9 × 10^7^

a The field-induced change was estimated by applying a field strength of 0.5 MV cm^–1^ in all cases.

**Figure 3:**
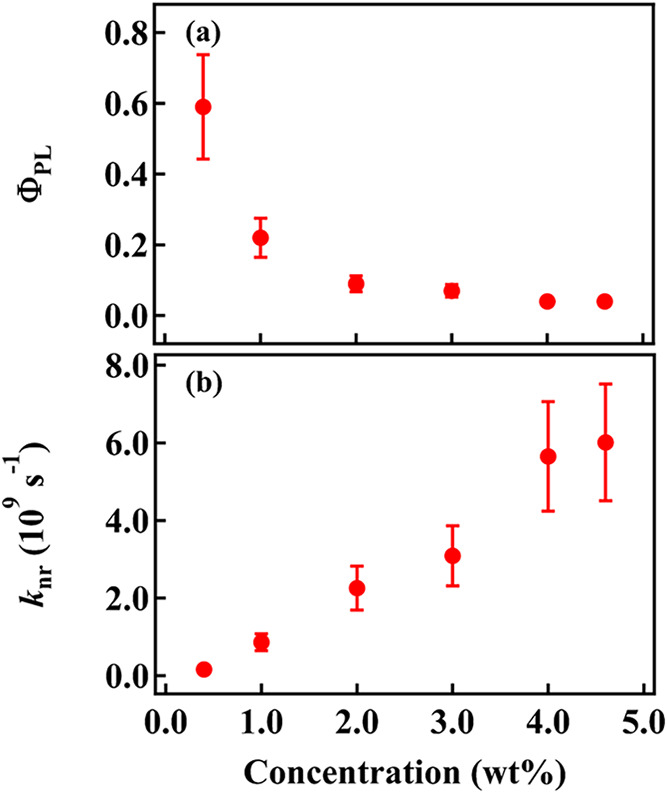
Plots of the PL quantum yield (a) and nonradiative rate constant (b) as a function of the concentration of FL in the PVA film.

### Electric Field Effect on Absorption Spectra of Fluorescein in PVA Film

The E-A spectra of FL in PVA film with different FL concentrations were measured at the second harmonic of the modulation frequency of the applied electric field. Representative E-A spectra for FL:PVA film containing 0.4 and 4.6 wt % FL are shown in Figure 4, while the E-A spectra of FL:PVA film observed for the intermediate FL concentrations are provided in SI (Figures S2 and S3). All measurements were carried out at an applied field strength of 0.5 MV cm^–1^. As the strength of the applied field increased, the amplitude of the E-A signal increased proportionally to the square of the field strength, as shown in Figures 4 and S2, S3, in agreement with the quadratic Stark effect. This behavior confirms that the observed E-A response originated from field-induced perturbations of the electronic states of the FL molecules. A weak feature observed at wavenumbers greater than 2.45 × 10^4^ cm^–1^ is likely due to interference between the FL:PVA film and substrate, as evidenced by the lack of reproducibility with different samples. Since the PVA film has a wide band gap, it does not contribute to the E-A response, ensuring that the measured spectra originate solely from the embedded FL. Furthermore, the insulating nature of the PVA matrix provides a uniform dielectric environment, enabling the external electric field to effectively couple with and modulate the electronic structure of the FL molecules through the Stark effect.

**Figure 4:**
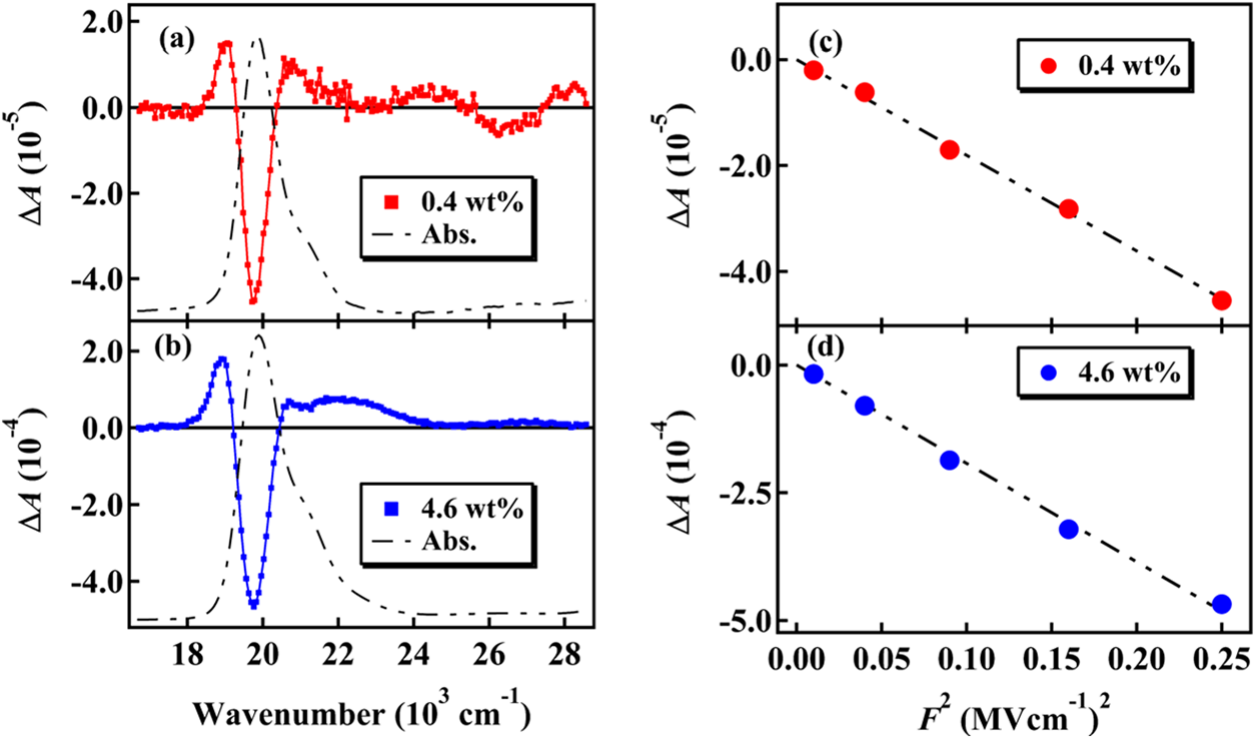
E-A spectra of FL:PVA film with FL concentrations of 0.4 wt % (a) and 4.6 wt % (b), measured with an applied electric field strength of 0.5 MV cm^–1^. The field strength dependence of the E-A signals, monitored at the minimum, is shown in (c) for the 0.4 wt % sample and in (d) for the 4.6 wt % sample. Absorption spectra are also shown in (a) and (b) by chain lines.

When an external electric field is applied, the energy of an electronic transition is modified by the difference in the electric dipole moment (Δμ) and molecular polarizability (Δα) between the ground and excited states, i.e., Δ*E* = −Δμ·*F* – *F*·Δα·*F*/2, where *F* is the applied electric field. To quantitatively analyze the observed E-A spectra, the field-induced change in the absorption intensity was simulated by using eq 1. The field-induced change in the absorbance at a given wavenumber (ν̅), i.e., Δ*A*(ν̅), can be expressed as a linear combination of the zeroth, first, and second derivatives of the absorption spectrum *A*(ν̅):^27–30^

1 $$\Delta A\left(\mathrm{\nu ̅}\right)={\left(\mathrm{fF}\right)}^{2}\left[\delta A\left(\mathrm{\nu ̅}\right)+\theta \mathrm{\nu ̅}\frac{d}{d\mathrm{\nu ̅}}\left(\frac{A\left(\mathrm{\nu ̅}\right)}{\mathrm{\nu ̅}}\right)+\eta \mathrm{\nu ̅}\frac{d^{2}}{d{\mathrm{\nu ̅}}^{2}}\left(\frac{A\left(\mathrm{\nu ̅}\right)}{\mathrm{\nu ̅}}\right)\right]$$

Here, *f* is the internal field factor, and *F* is the magnitude of the externally applied electric field. The coefficients δ, θ, and η are fitting parameters that represent distinct physical contributions to the E-A spectrum. The coefficient δ is associated with the field-induced change in the transition moment or oscillator strength, while coefficients θ and η correspond to the spectral shift and broadening, respectively, which mainly result from the change in molecular polarizability and electric dipole moment, i.e., Δα and Δμ. The coefficients θ and η are directly related to the molecular parameters as

2 $$\theta =\frac{\Delta \mathrm{\alpha ̅}}{2\mathrm{hc}};\eta =\frac{{\left|\Delta \mu \right|}^{2}}{6h^{2}c^{2}}$$

where Δα̅ denotes the trace of Δα, *h* is the Planck’s constant, and *c* is the speed of light. These relations allow the quantitative extraction of |Δμ| and Δα̅ from the experimentally measured E-A spectra.

Figures 5 and S4, S5 show the simulated E-A spectra of the FL:PVA film obtained by fitting the experimental data with three Gaussian bands of the absorption spectra (G1–G3), except for 0.4 wt %, where two Gaussian bands (G1 and G2) were enough to adequately reproduce the spectral features, indicating contributions from multiple vibronic transitions. Quantitative analysis of the E-A spectra yields |Δμ| and Δα̅ of the FL:PVA film. These values, as well as coefficients δ, θ, and η, are summarized in Table S1. The magnitudes of Δμ and Δα̅ estimated for each of the G1, G2, and G3 bands are plotted in Figure 6, as a function of FL concentration. In the analysis, the G1, G2, and G3 bands are located at 1.98 × 10^4^ cm^–1^ (505 nm), 2.08 × 10^4^ cm^–1^ (481 nm), and 2.25 × 10^4^ cm^–1^ (444 nm), respectively. The value of |Δμ| is ∼3 D for G1 and ∼0.6 D for G2 and G3 at any FL concentration, and Δα̅ is estimated to be 1.2 ± 1.0 Å^3^ for G1, −0.5 ± 0.2 Å^3^ for G2, and 0.7 ± 0.5 Å^3^ for G3. As shown in Figure 6, Δα̅ for G1 tends to increase with FL concentration, Δα̅ for G2 is nearly independent of FL concentration, and Δα̅ for G3 tends to decrease with FL concentration.

**Figure 5:**
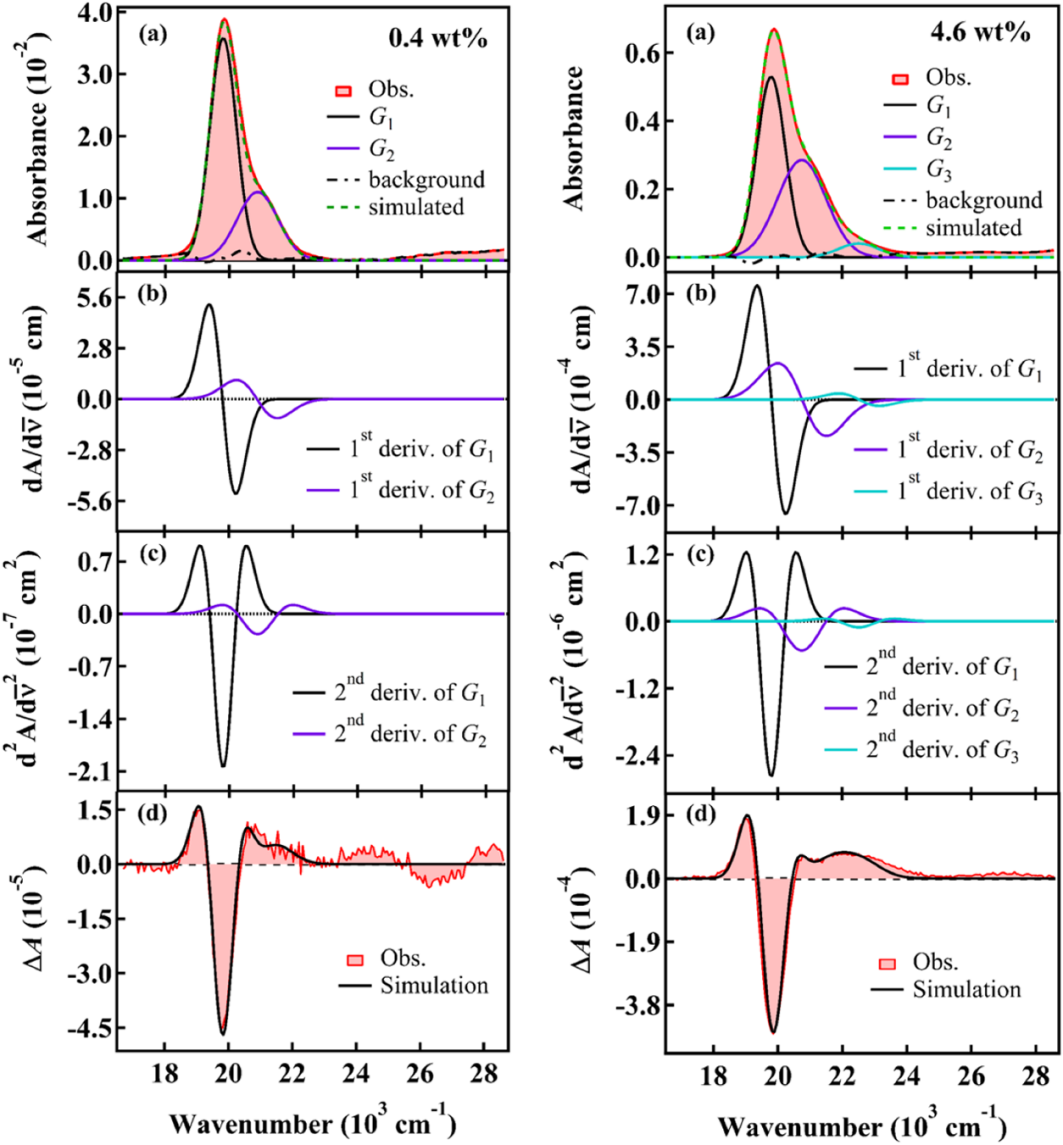
Analysis of the E-A spectra of FL:PVA film with FL concentrations of 0.4 wt % (left) and 4.6 wt % (right). Absorption spectra and their decomposed Gaussian bands (a), first derivatives of the Gaussian bands (b), second derivatives of the Gaussian bands (c), and simulated E-A spectra with the observed spectra (d).

**Figure 6:**
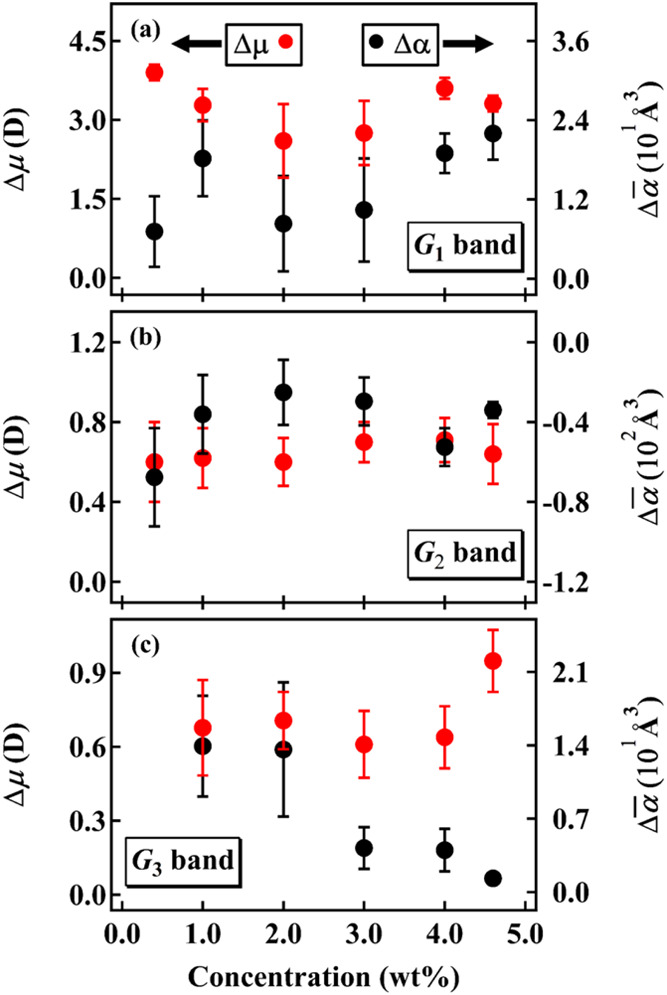
Plots of the magnitude of Δμ (left, red circles) and Δα̅ (right, black circles) estimated from the E-A spectra for the G1 band (a), G2 band (b), and G3 band (c), as a function of FL concentration in PVA.

Even when the absorption bands correspond to transitions to different vibronic levels of the same electronically excited state, the values of |Δμ| and Δα̅ may differ from each other. In fact, vibrational Stark spectra, where optical transitions occur to different vibrational states belonging to the same ground electronic state, showed that the different vibrational transitions give different values of |Δμ| and Δα̅, as we reported in α-helical polypeptide,^31^ indicating that Stark spectroscopy depends not only on the electronic transition but also on the vibrational level to which the optical transition occurs. Then, G1 and G2 bands may be considered vibronic bands belonging to optical transitions to the same electronic state. The mirror-image relationship between the absorption and fluorescence spectra (Figure S1) seems to support this assignment. However, another possibility must also be considered regarding the assignment of the G1 and G2 bands. The density functional theory (DFT) data for four fluorescein species, i.e., the dianion, the monoanion, the neutral, and the cation forms were reported by Batistela et al.^32^ They suggested two representative signals in the dianion form, which is related to the present work, i.e., an intense band at 437 nm and a weak band at 408 nm with an absorption intensity ratio of 0.60:0.11, which corresponds to the HOMO → LUMO transition and HOMO – 5 → LUMO transition, respectively. According to that study, these bands are considered to correspond to the observed bands at 490 and 470 nm, which correspond to the G1 and G2 bands in the present absorption spectra. The energy separation between these two bands obtained in the DFT calculation, i.e., ∼1630 cm^–1^, is not significantly different from the separation between G1 and G2, i.e., 1000 cm^–1^. The calculated bands exhibited a large blue shift of more than 50 nm compared to the observed bands. It is not clear why the location of the peak position in the DFT data was so different from the observed one, but these results may indicate the need to consider two possibilities for the assignment of the G1 and G2 bands: transitions between different vibrational states within the same electronic state and transitions between different electronic states, which remains a task for future work.

For the G3 band, which reflects the presence of aggregates at high concentrations, the increase in |Δμ| and decrease in Δα̅ with increasing FL concentration suggest that the excited state associated with the aggregate acquires *N* enhanced charge-separated character while simultaneously becoming electronically less deformable with increasing FL concentration.

As mentioned earlier, the zeroth derivative term in eq 1 corresponds to the field-induced change in the absorption intensity. Since the radiative decay rate constant (*k*_r_) in the emitting state is proportional to the absorption intensity, the electric field-induced change in *k*_r_, Δ*k*_r_, can be estimated using the following equation:

3 $$\Delta A/A=\Delta k_{r}/k_{r}$$

where Δ*A* and *A* correspond to ∫Δ*A*(ν̅)­dν̅ and ∫*A*(ν̅)­dν̅, respectively, and *A* and *k*_r_ are the values at the zero field. The negative values of δ in eq 1, as well as the field-induced decrease in absorption intensity (Figures 4 and S2, S3), indicate that *k*_r_ decreases with the application of the electric field, that is, Δ*k*_r_ is negative. Here, Δ*A* and *A* were obtained at each concentration by integrating the whole of E-A and absorption spectra, respectively, without distinguishing between the G1, G2, and G3 bands. The results are shown in Table 1 and Figure 7, which indicate that the magnitude of Δ*k*_r_ is very large at very low concentration, but it decreases with increasing FL concentration in PVA. When the results of the analysis of the E-A spectra by using eq 3, which are presented in Table S1, are carefully observed, the field-induced decrease in *k*_r_, i.e., negative values of coefficient δ, is only for G1 and G2 bands, whereas the G3 band shows a field-induced increase in the absorption intensity at concentrations above 1.0 wt %, indicating that *k*_r_ increases with the application of an electric field for the G3 absorption band, in contrast with G1 and G2.

**Figure 7:**
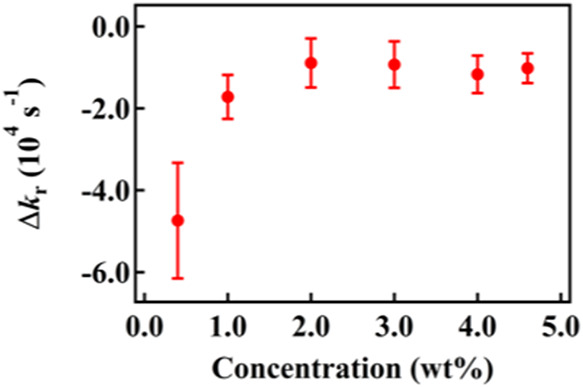
Plots of Δ*k*_r_ of FL doped in PVA film as a function of FL concentration.

As mentioned above, the three bands, i.e., G1–G3, were necessary to reproduce the E-A spectra at FL concentrations higher than 1.0 wt %, whereas the E-A spectra at 0.4 wt % could be simulated by considering two bands, G1 and G2. The absence of the G3 band at 0.4 wt % indicates that the G3 band is assigned to the absorption band of an aggregate of FL produced in PVA film at high concentrations of FL. This is supported by the opposite behavior of the electric field effects on *k*_r_; that is, δ is negative for G1 and G2, but positive for G3 at any FL concentration, as mentioned above. With the application of external electric fields, therefore, the absorption intensities of G1 and G2 decrease, whereas the absorption intensity of G3 increases. The large magnitude of the field-induced change in *k*_r_ observed at low concentrations is due to the direct Stark perturbation of isolated FL monomers, resulting from the large decrease of the absorption intensity in the presence of an applied electric field, i.e., in the G1 and G2 bands. As the FL concentration increases, aggregation becomes more pronounced, and the field-induced enhancement of the absorption intensity of the aggregate, i.e., G3 band, increases, though the absorption intensity of the G3 band is very small. When the field-induced change in the total absorption intensity is monitored, the magnitude of the field-induced decrease in the absorption intensity becomes smaller with increasing concentration because the field-induced decrease in the G1 and G2 bands is canceled by the field-induced increase of the absorption intensity of G3. These results clearly indicate that the field-induced change in *k*_r_ is opposite for the monomer and aggregates, resulting in a decrease of the magnitude of the field-induced change in *k*_r_ with increasing FL concentration.

### Electric Field Effects on the Photoluminescence (PL) Spectra of Fluorescein in a PVA Film

The influence of an external electric field on the PL spectra of FL in a PVA film was also examined. Figure 8 shows the representative E-PL spectra of the FL:PVA film containing 0.4 and 4.6 wt % FL with an applied field strength of 0.5 MV cm^–1^. The excitation was done at 470 nm, where the field-induced change in the absorption intensity was negligible, ensuring that the observed E-PL response resulted primarily from the field-induced modulation of the emission process rather than changes in the optical absorption intensity. The E-PL spectra of the FL:PVA film with different FL concentrations are shown in Figures S7 and S8. The PL and E-PL spectra were recorded simultaneously to ensure reliable comparison. All of the FL:PVA film exhibited a pronounced negative E-PL signal in the main emission band. This negative feature indicates a decrease in the PL intensity upon the application of an electric field.

**Figure 8:**
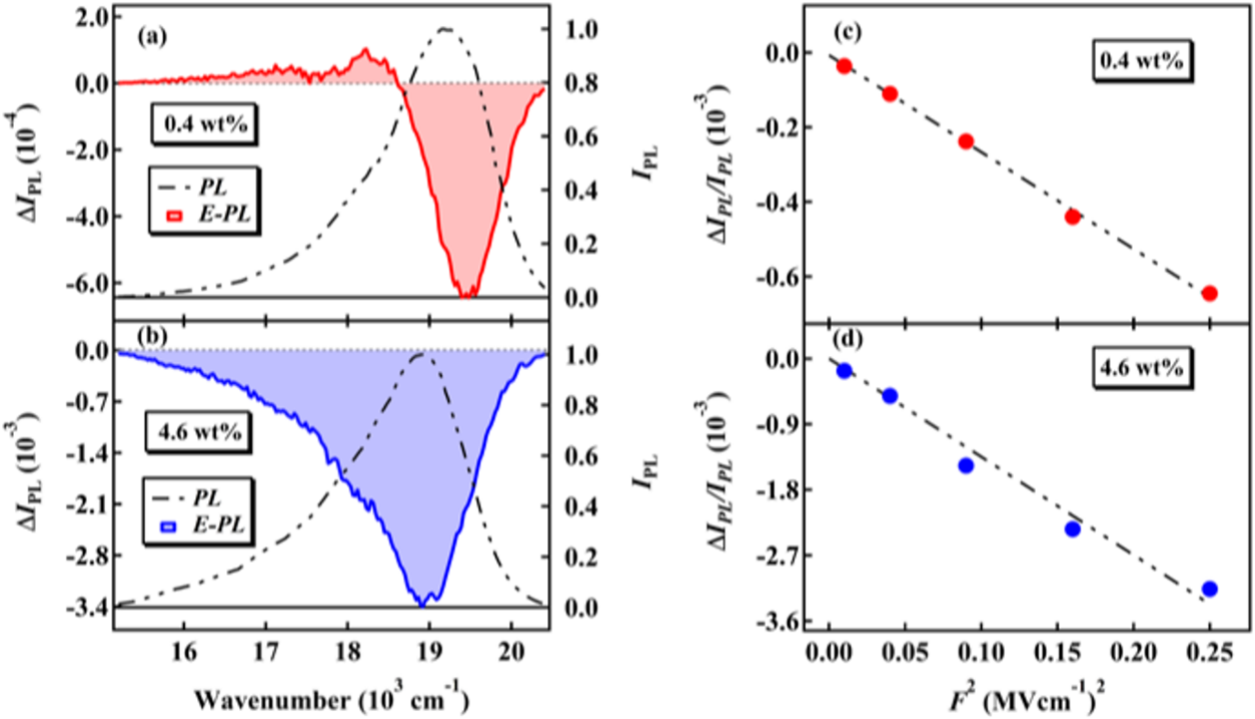
E-PL spectra of FL:PVA film measured with a field strength of 0.5 MV cm^–1^ for the samples with FL concentrations of 0.4 wt % (a) and 4.6 wt % (b), together with the PL spectra. Applied field strength dependence of the field-induced change in the PL intensity relative to the PL intensity (Δ*I*_PL_/*I*_PL_) for the samples with FL concentrations of 0.4 wt % (c) and 4.6 wt % (d), monitored at the minimum of the E-PL signal.

The field-induced change in PL intensity (Δ*I*_PL_) is proportional to the square of the applied field strength (see Figure 8 and S7, S8), indicating that the observed response is governed by quadratic field effects, consistent with the second-order Stark effect. In such a case, the E-PL intensity can be expressed by the following equation, as in the case of E-A spectra:^33,34^

4 $$\Delta I_{\mathrm{PL}}\left(\mathrm{\nu ̅}\right)={\left(\mathrm{fF}\right)}^{2}\left[\delta^{\prime }I_{\mathrm{PL}}\left(\mathrm{\nu ̅}\right)+\theta^{\prime }\nu^{3}\frac{d}{d\mathrm{\nu ̅}}\left(\frac{I_{\mathrm{PL}}\left(\mathrm{\nu ̅}\right)}{\overset{®}{\nu^{3}\;}}\right)+\eta^{\prime }{\mathrm{\nu ̅}}^{3}\frac{d^{2}}{d{\mathrm{\nu ̅}}^{2}}\left(\frac{I_{\mathrm{PL}}\left(\mathrm{\nu ̅}\right)}{{\mathrm{\nu ̅}}^{3}}\right)\right]$$

Here, Δ*I*_PL_(ν̅) = *I*_PL_^*F*^(ν̅) – *I*_PL_^0^(ν̅), where *I*_PL_^*F*^(ν̅) and *I*_PL_^0^(ν̅) are the PL intensities at wavenumber ν̅ in the presence and absence of an applied electric field, *F*, respectively. The first term in eq 4 corresponds to the field-induced change in PL intensity, i.e., field-induced quenching or enhancement. The observed field-induced decrease in the PL intensity is interpreted in terms of this term. The θ′ and η′ in eq 4 correspond to θ and η in eq 1, and the change in molecular polarizability and electric dipole moment following emission can be deduced from these terms, respectively. In fact, the E-PL spectrum at 0.4 wt % was different in shape from the PL spectrum and attempted to reproduce with eq 4, as shown in Figure 9. In the simulation, an emission spectrum similar in shape to the observed PL spectrum was produced by combining three Gaussian profiles to obtain smooth derivative shapes. When only one band is employed for simulation, the observed E-PL spectra cannot be accurately reproduced, especially in the higher energy region, implying that the PL spectra are composed of several bands with different values of |Δμ| and Δα̅, similar to the E-A spectra. Based on the simulation shown in Figure 9, the magnitudes of Δα̅ and |Δμ| are estimated to be 49 Å^3^ and 2.4 D, respectively, from the contribution of the second and third terms in eq 4. The obtained value of |Δμ| is smaller than that estimated from the analysis of the E-A spectra for the G1 band, ∼3 D, probably because the emitting state is different from the photoexcited state owing to the vibrational relaxation.

**Figure 9:**
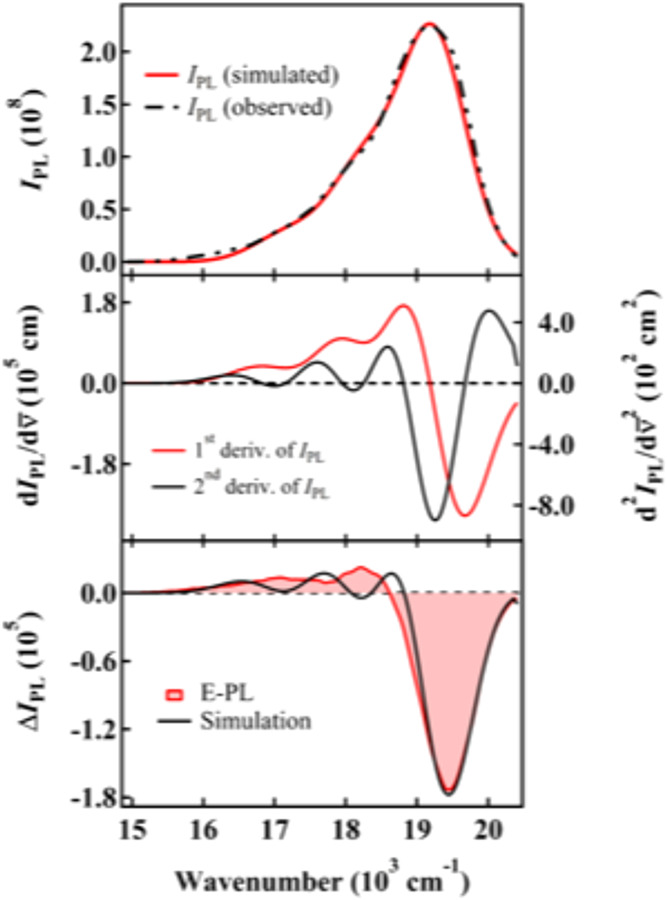
Simulation of the E-PL spectrum of the FL:PVA film with a FL concentration of 0.4 wt %. The PL spectrum is obtained as a sum of three Gaussian bands to obtain smooth derivative shapes (top), the first and second derivatives of the PL spectrum (middle), and the simulated E-PL spectrum and the observed E-PL spectrum (bottom). The observed PL spectrum is also superimposed by a black chain line (top).

With increasing FL concentration, the E-PL spectra became nearly the same in shape as the PL spectra (Figures 8 and S7, S8), indicating that the contribution of the first term in eq 4 became dominant, that is, the magnitude of the field-induced quenching of PL became larger with increasing FL concentration. To quantify the extent of electric-field-induced emission modulation, the ratio Δ*I*_PL_/*I*_PL_ was evaluated at each concentration, reflecting directly the degree of PL quenching or enhancement induced by the applied field. Note that Δ*I*_PL_ and *I*_PL_ correspond to ∫Δ*I*_PL_(ν̅)­dν̅ and ∫*I*_PL_^0^(ν̅)­dν̅, respectively. By using these values, the field-induced change in the PL quantum yield (ΔΦ_PL_) can be estimated using the relation $\frac{\Delta \Phi_{\mathrm{PL}}}{\Phi_{\mathrm{PL}}}=\frac{\Delta I_{\mathrm{PL}}}{I_{\mathrm{PL}}}$ The results are shown in Table 1 and Figure S9, which indicate that the field-induced quenching of PL increased with increasing FL concentration and became nearly constant at high concentrations.

In principle, the application of an electric field may affect both the radiative decay rate constant (*k*_r_) and the nonradiative decay rate constant (*k*_nr_) in the emitting state. As mentioned earlier, *k*_r_ decreased with the application of an electric field at any concentration, except for the G3 band. The field-induced change in *k*_nr_ (Δ*k*_nr_) can also be estimated using the following equation:

5 $$\Delta k_{\mathrm{nr}}=\left(I_{\mathrm{PL}}/I_{\mathrm{PL}}^{F}\right)\left\{\left(k_{r}+{\Delta k}_{r}\right)/k_{r}\tau \right\}-\left(\Delta k_{r}+\frac{1}{\tau }\right)$$

as

$$\Phi_{\mathrm{PL}}^{F}/\Phi_{\mathrm{PL}}=I_{\mathrm{PL}}^{F}/I_{\mathrm{PL}}=\left[\left(k_{r}+{\Delta k}_{r}\right)/{(k}_{r}+{\Delta k}_{r}+k_{\mathrm{nr}}+{\Delta k}_{\mathrm{nr}})\right]/\left\{k_{r}/{(k}_{r}+k_{\mathrm{nr}})\right\}$$

where Φ_PL_^*F*^ and *I*_PL_^*F*^ represent the PL quantum yield and PL intensity, respectively, in the presence of an external electric field. The obtained Δ*k*_nr_ values are presented in Table 1. Plots of Δ*k*_nr_ as a function of FL concentration are also shown in Figure 10. Δ*k*_nr_ increases monotonically and rapidly with increasing FL concentration, indicating that the field-induced enhancement of nonradiative processes from the fluorescent species of FL to nonemissive species becomes more efficient with increasing FL concentration in the PVA film. If the fluorescence quenching is due to static quenching, that is, if the FL that makes up the aggregate does not show fluorescence, the fluorescence lifetime is usually independent of the concentration. On the other hand, the fluorescence lifetime becomes shorter during dynamic quenching, where the emission property depends on the donor–acceptor distance. The present results of the concentration-dependent *k*_nr_ remind us of the dynamic quenching in FL fluorescence. The concentration dependence of Δ*k*_nr_ indicates that both *k*_nr_ and Δ*k*_nr_ depend on the donor–acceptor distance, i.e., the distance between the FL monomer and aggregates, indicating that aggregate-mediated relaxation processes are very sensitive to the external electric field.

**Figure 10:**
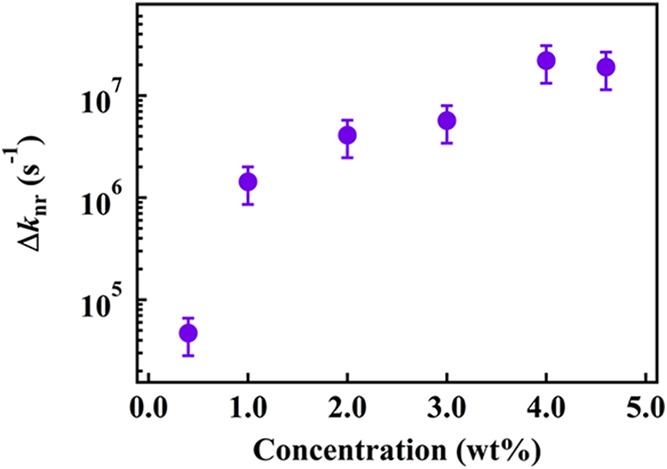
Plots of the electric field-induced change in *k*_nr_ (Δ*k*_nr_) for the FL:PVA film as a function of the FL concentration.

With an increase in the FL concentration, the average intermolecular distance decreases, favoring molecular aggregation in the PVA matrix. Energy transfer between excited species of FL and another FL, i.e., fluorescence resonance energy transfer (FRET), can be considered to occur more efficiently with increasing FL concentration. However, it is unlikely that simple FRET between the fluorescence FL and nonexcited FL monomer induced a decrease of the fluorescence lifetime and fluorescence quantum yield, because both donor and acceptor molecules are considered to have similar fluorescence behavior. Rather, additional nonradiative processes for nonemissive aggregates of FL, leading to aggregation-induced quenching, may have to be considered. This is reflected by a systematic increase in *k*_nr_ and a concurrent reduction in the PL lifetime with increasing FL concentration.

As mentioned in the analysis of the E-A spectra, the third band, i.e., G3, must be considered to simulate the EA spectra at concentrations above 1.0 wt %. At a low concentration of 0.4 wt %, the G3 band was not necessary to simulate the E-A spectra, implying that G3 is assigned to the band caused by an aggregate. The fact that the G3 band is located in a higher energy region than G1 and G2 suggests that the G3 band is assigned to the H-aggregate, which is known to give a blue-shifted absorption spectrum compared to the isolated monomer, along with weak or no fluorescence.^35–37^ It is considered that the nonradiative process from the fluorescent state of the FL monomer to the nonemissive H-aggregates gives a large *k*_nr_ as well as Δ*k*_nr_ at high concentrations of FL in PVA film. The PL spectra of FL in PVA film at 2.0 and 3.0 wt % showed the same spectra upon excitation at 506, 493, and 445 nm, as shown in Figure S10, which corresponds to the excitation into G1, G2, and G3, respectively. If excitation into the G3 band leads to relaxation to the nonemissive state (lowest energy level of the H-aggregates), it is reasonable to consider that the PL spectra observed with excitation at 445 nm are caused by excitation of the FL monomer and that H-aggregates are nonemissive. The highly efficient electric field effect on *k*_nr_ may imply that a charge transfer process is involved in this nonradiative process.

## Conclusion

The fluorescence lifetime, fluorescence quantum yield, and nonradiative decay rate constant of fluorescein (FL) doped in poly­(vinyl alcohol) (PVA) film were determined for different concentrations of FL in PVA film. It is found that the lifetime and quantum yield decrease rapidly with increasing FL concentration because the nonradiative decay rate at the emitting state of FL in the PVA film increases rapidly with increasing FL concentration. Both E-A and E-PL spectra were obtained for FL at different concentrations in PVA film. Based on the analysis of the E-A spectra, the magnitudes of the change in the electric dipole moment and polarizability following photoexcitation, as well as the field-induced change in the radiative decay rate constant, were estimated for FL-doped PVA film at each concentration of FL. At very low concentrations, the E-A spectra could be analyzed by assuming that the absorption band was composed of two Gaussian bands (G1 and G2 bands), while the absorption bands composed of three Gaussian bands (G1–G3 bands) were necessary for the analysis of the E-A spectra at high FL concentrations. The presence of the G3 band located at a higher energy region than the G1 and G2 bands was confirmed only at high FL concentrations, suggesting the formation of H-aggregates in PVA film at high FL concentrations. E-PL measurements show the field-induced quenching of the fluorescence of FL, resulting from the field-induced increase of the nonradiative decay rate constant (*k*_nr_). The field-induced increase of *k*_nr_, i.e., Δ*k*_nr_. is found to increase drastically with increasing FL concentration in PVA, which probably results from the nonemissive energy dissipation to the H-aggregates.

## Supplementary Material



## Notes

Absorption and PL spectra of fluorescein (FL) in solution; E-A spectra of FL:PVA film and applied field strength dependence of the E-A signal at FL concentrations of 1.0, 2.0, 3.0, and 4.0 wt %; analysis of the E-A spectra of the FL:PVA film at FL concentrations of 1.0, 2.0, 3.0, and 4.0 wt %; plots of Δ*A*/*A* as a function of FL concentration; E-PL spectra of FL:PVA film and applied field strength dependence of the E-PL signal at FL concentrations of 1.0, 2.0, 3.0, and 4.0 wt %; applied electric field-induced change in the PL intensity of FL:PVA film as a function of FL concentration; excitation wavelength dependence of PL spectra; analysis data of the E-A spectra of FL:PVA film at different FL concentrations (PDF)

## References

[1] Maiya S., Martis G. J., Shetty N. S., Gaonkar S. L. (2025). Organic Fluorescent
compounds: A Review of Synthetic Strategies and Emerging Applications. Discovery Appl. Sci..

[2] Sun N., Cai Y., Cui T., Yang W., Hu Y. (2023). Organic Dye Fluorescent
Fibers for Wearable Devices. Opt. Mater..

[3] Afanas’ev D. A., Davidenko N. A., Davidenko I. I., Ibraev N. Kh., Mokrinskaya E. V., Studzinskii S. L., Pavlov V. A., Tonkopieva L. S., Chuprina N. G. (2015). Photovoltaic Properties of Films of Polyvinyl Alcohol
– Xanthene Dye Composites. High Energy
Chem..

[4] Pepe G., Cole J. M., Waddell P. G., Griffiths J. R. D. (2016). Molecular
Engineering of Fluorescein Dyes as Complementary Absorbers in Dye
Co-Sensitized Solar Cells. Mol. Syst. Des. Eng..

[5] Abdullah R. M., Badran H. A., Abdul-Hail R. C. (2024). Electrical,
Thermal, Lens and Optical
Study of Fluorescein Film for Application as Organic Photovoltaic
Devices. J. Fluoresc..

[6] da
Silva B. H. S. T., Bregadiolli B. A., Graeff C. F. d. O., da Silva-Filho L. C. (2017). NbCl_5_-Promoted Synthesis of Fluorescein
Dye Derivatives: Spectroscopic and Spectrometric Characterization
and Their Application in Dye-Sensitized Solar Cells. ChemPlusChem.

[7] Abutalib M. M., Shkir M., Yahia I. S., Alfaify S., El-Naggar A. M., Ganesh V. (2016). Thickness Dependent Optical Dispersion and Nonlinear
Optical Properties of Nanocrystalline Fluorescein Dye Thin Films for
Optoelectronic Applications. Optik.

[8] Saleh B. R., Naser B. A., Salman Z. N. (2023). Non-Linear
optical Properties for
Thin Films of Fluorescein Organic Laser Dyes Doped with Polyvinyl
Alcohol Polymer and Al2O3 Nanoparticles. Eng.
Proc..

[9] Jin J.-Y., Kim H.-G., Hong C.-H., Suh E.-K., Lee Y.-S. (2007). White Light
Emission from a Blue LED, Combined with a Sodium Salt of Fluorescein
Dye. Synth. Met..

[10] Al-Aqmar D. M., Abdelkader H. I., Abou Kana M. T. H. (2015). Spectroscopic Properties and Amplified
Spontaneous Emission of Fluorescein Laser Dye in Ionic Liquids as
Green Media. Opt. Mater..

[11] Sjöback R., Nygren J., Kubista M. (1995). Absorption
and Fluorescence Properties
of Fluorescein. Spectrochim. Acta, Part A.

[12] Tatar P., Kacik D., Tarjanyi N. (2016). Fluorescein
Filled Photonic Crystal
Fiber Sensor for Simultaneous Ultraviolet Light and Temperature Monitoring. Opt. Fiber Technol..

[13] Urano Y., Kamiya M., Kanda K., Ueno T., Hirose K., Nagano T. (2005). Evolution of Fluorescein as a Platform
for Finely Tunable
Fluorescence Probes. J. Am. Chem. Soc..

[14] Margulies D., Melman G., Shanzer A. (2005). Fluorescein
as a Model Molecular
Calculator with Reset Capability. Nat. Mater..

[15] Yan F., Fan K., Bai Z., Zhang R., Zu F., Xu J., Li X. (2017). Fluorescein
Applications as Fluorescent Probes for the Detection
of Analytes. TrAC, Trends Anal. Chem..

[16] Naderi F., Farajtabar A. (2016). Solvatochromism
of Fluorescein in Aqueous Aprotic Solvents. J. Mol. Liq..

[17] Martin M. M., Lindqvist L. (1975). The pH Dependence of Fluorescein
Fluorescence. J. Luminesc..

[18] Klonis N., Sawyer W. H. (1996). Spectral Properties
of the Prototropic Forms of Fluorescein
in Aqueous Solution. J. Fluoresc..

[19] Sasai R., Morita M. (2017). Luminous Relative Humidity
Sensing by Anionic Fluorescein
Dyes Incorporated into Layered Double Hydroxide/1-Butanesulfonate
Hybrid Materials. Sens. Actuators, B.

[20] Patil V., Padalkar V., Sekar N. (2015). Environment-Sensitive
Benzoxazole
Based Fluorescein Derivatives: Synthesis and Application to the Design
of On-OFF Fluorescent Chemosensors for Microenvironment. J. Luminesc..

[21] Lee J. H., Jung D.-Y., Kim E., Ahn T. K. (2014). Fluorescein
Dyes
Intercalated Layered Double Hydroxides for Chemically Stabilized Photoluminescent
Indicators on Inorganic Surfaces. Dalton Trans..

[22] Li F., Wang X., Zhu M., Liu D., Liu D., Zhao J. (2022). Fluorescence Properties of Fluorescein
and Rhodamine Supported on
Alumina Nanowire Films. Ceram. Int..

[23] Atvars T. D. Z., Bortolato C. A., Dibbern-Brunelli D. (1992). Electronic Absorption and Fluorescence
Spectra of Xanthene Dyes in Polymers. J. Photochem.
Photobiol., A.

[24] Awasthi K., Wang C.-Y., Fathi A., Narra S., Diau E. W.-G., Ohta N. (2017). Anisotropic Electric Field Effect on Photoluminescence
of CH3NH3PbI3 Perovskite Sandwiched between Conducting and Insulating
FilmsJJ. Phys. Chem. C.

[25] Rana S., Awasthi K., Bhosale S. S., Diau E. W.-G., Ohta N. (2019). Temperature
Dependent Electroabsorption and Electrophotoluminescence and Exciton
Binding Energy in MAPbBr_3_ Perovskite Quantum Dots. J. Phys. Chem. C.

[26] Strickler S. J., Berg R. A. (1962). Relationship Between Absorption Intensity
and Fluorescence
Lifetime of Molecules. J. Chem. Phys..

[27] Liptay W. (1974). Dipole Moments
and Polarizability of Molecules in Excited Electronic States. Excited States.

[28] Bublitz G. U., Boxer S. G. (1997). Stark Spectroscopy: Applications in Chemistry, Biology
and Materials Science. Annu. Rev. Phys. Chem..

[29] Jalviste E., Ohta N. (2007). Theoretical Foundation
of Electroabsorption Spectroscopy: Self-Contained
Derivation of the Basic Equations with the Direction Cosine Method
and the Euler Angle Method. J. Photochem. Photobiol.,
C.

[30] Rana S., Tsai I.-H., Awasthi K., Narra S., Bhosale S. S., Ma D., Diau E. W.-G., Ohta N. (2025). Morphology-Dependent Charge-Separated
Character in the Photoexcited States of the Photocatalytic Bismuth
Oxyiodide nanocrystals and Nanosheets: Implications for Photodynamic
Therapy. ACS Appl. Nano Mater..

[31] Nakabayashi T., Ohta N. (2005). Electric Field Effects
on IR Absorption of α-helical polypeptide. Chem. Lett..

[32] Batistela V. R., Cedran J., da C., de Oliveira H. P. M., Scarminio I. S., Ueno L. T., Machado A. E., da H., Hioka N. (2010). Protolyc Fluorescein Species Evaluated Using Chemometry and DFT Studies. Dyes Pigm..

[33] Umeuchi S., Nishimura Y., Yamazaki I., Murakami H., Yamashita M., Ohta N. (1997). Electric Field Effects on Absorption and Fluorescence Spectra of
Pyrene Doped in a PMMA Polymer Film. Thin Solid
Films.

[34] Ohta N. (2002). Electric Field
Effects on Photochemical Dynamics in Solid Films. Bull. Chem. Soc. Jpn..

[35] Kasha M., Rawls H. R., Ashraf El-Bayoumi M. (1965). The Exciton Model in Molecular Spectroscopy. Pure Appl. Chem..

[36] Emerson E. S., Conlin M. A., Rosenoff A. E., Norland K. S., Rodriguez H., Chin D., Bird G. R. (1967). The Geometrical
Structure and Absorption
Spectrum of a Cyanine Dye Aggregate. J. Phys.
Chem. A.

[37] Sundström V., Gillbro T. (1985). Excited State Dynamics and Photophysics of Aggregated
Dye Chromophores in Solution. J. Chem. Phys..

