# Supplementary material for: Probing Excited-State Electronic Structure and Dynamics of Fluorescein Doped in PVA Film Using Electroabsorption and Electrophotoluminescence Spectroscopy
Source: J Phys Chem B. 2026 Jul 27;130(31):8103–12. doi: 10.1021/acs.jpcb.6c02869 (PMC13455059; doi:10.1021/acs.jpcb.6c02869)
Supplement: Supplementary file 1 [file jp6c02869_si_001.pdf]

Supporting Information

Probing Excited-State Electronic Structure and Dynamics of Fluorescein Doped in PVA Film Using Electroabsorption and Electrophotoluminescence Spectroscopy

Shailesh Rana,[1,2] Rong-Lin Jhang,[1] Kamlesh Awasthi,[1,3] Nobuhiro Ohta[1,3,*]

[1]Department of Applied Chemistry and Institute of Molecular Science, National Yang Ming Chiao Tung University, 1001 Ta-Hsueh Road, Hsinchu 300093, Taiwan

[2]Centre of Energy Research, Graphic Era Deemed to be University, Bell Road, Clement Town, Dehradun, 248002, India

[3]Center for Emergent Functional Matter Science, National Yang Ming Chiao Tung University, 1001 Ta-Hsueh Road, Hsinchu 300093, Taiwan

*Email: nohta@nycu.edu.tw

Figure S1: Absorption and PL spectra of fluorescein (FL) in solution

Figure S2: E-A spectra of FL:PVA films having a FL concentration of 1.0 wt% and 2.0 wt%, and applied field strength dependence of the E-A signal

Figure S3: E-A spectra of FL:PVA films having a FL concentration of 3.0 wt% and 4.0 wt%, and applied field strength dependence of the E-A signal

Figure S4: Analysis of the E-A spectra of FL:PVA film having a FL concentration of 1.0 wt% (left) and 2.0 wt% (right)

Figure S5: Analysis of the E-A spectra of FL:PVA film having a FL concentration of 3.0 wt% (left) and 4.0 wt% (right)

Figure S6: Plots of $\Delta A/A$ as a function of FL concentration

Figure S7: E-PL spectra of FL:PVA films having a FL concentration of 1.0 and 2.0 wt% and applied field strength dependence

Figure S8: E-PL spectra of FL:PVA films having a FL concentration of 3.0 and 4.0 wt% and applied field strength dependence

Figure S9: Applied electric field-induced change in PL intensity of FL:PVA films as a function of FL concentration

Figure S10: Excitation wavelength dependence of PL spectra of FL:PVA films

Table S1: The results of the analysis of the E-A spectra at different concentrations of FL

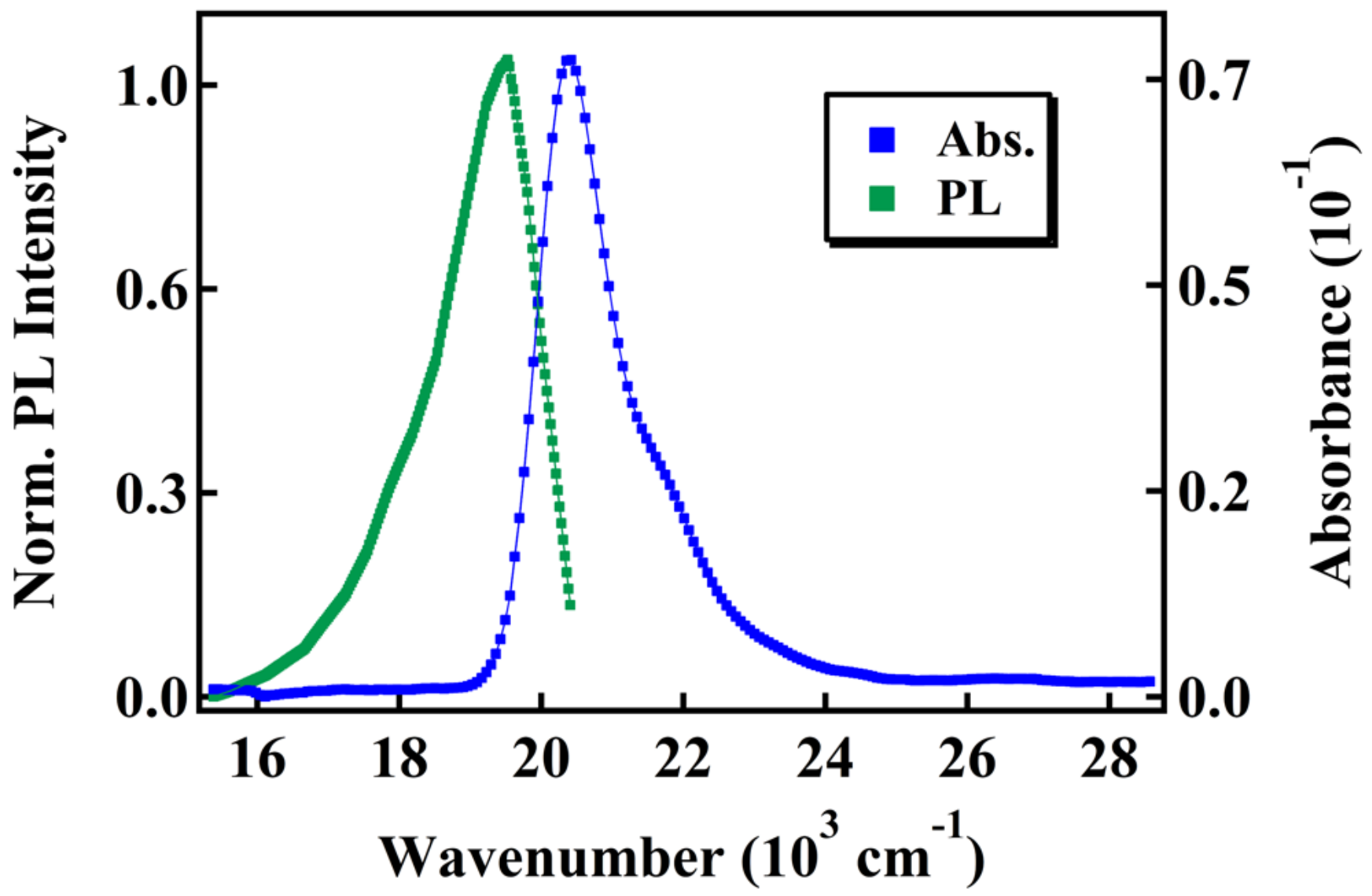

Figure S1. UV-Vis absorption (right axis) and PL (left axis) spectra of 1 μM fluorescein (FL) solution (0.1 M NaOH). The excitation wavelength for the PL measurement was 470 nm.

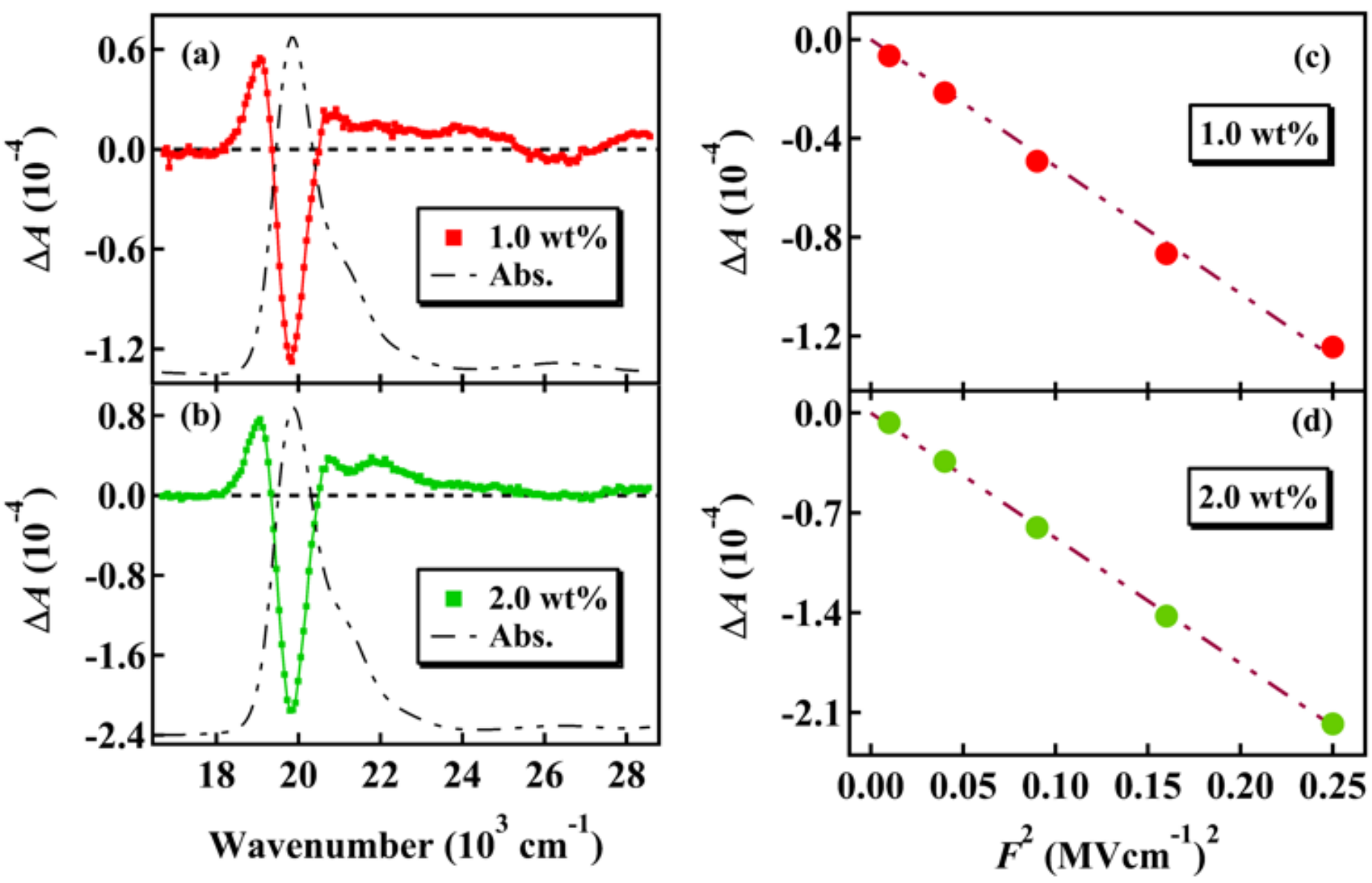

Figure S2. E-A spectra of FL:PVA film measured with FL concentrations of 1.0 wt% (a) and 2.0 wt% (b), measured with an applied electric field strength of 0.5 MVcm^{-1}. The field strength dependence of the E-A signals monitored at the minimum is shown in (c) for the 1.0 wt% sample and in (d) for 2.0 wt% sample. Absorption spectra are also shown in (a) and (b) by chain lines.

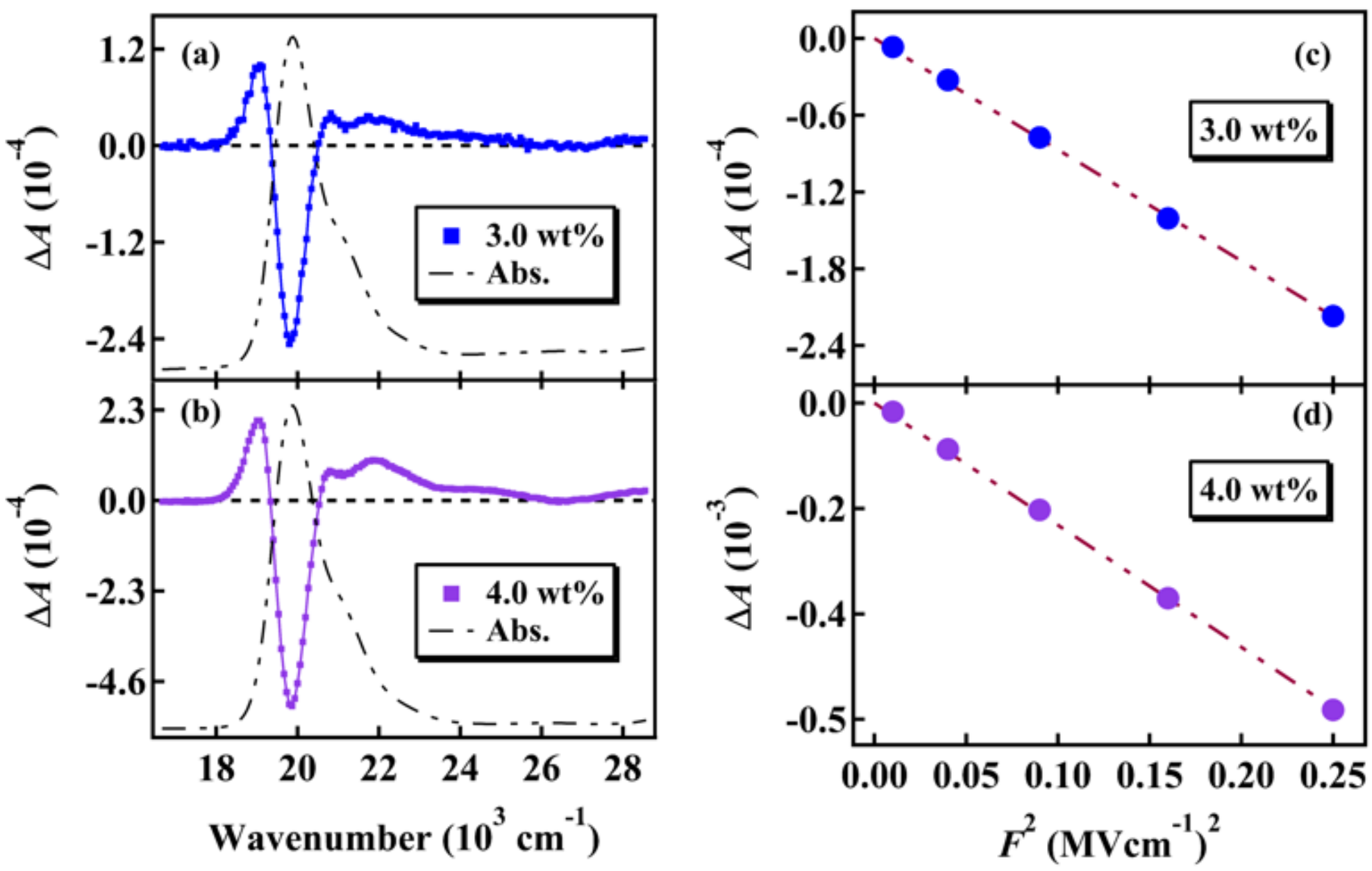

Figure S3. E-A spectra of FL:PVA film measured with FL concentrations of 3.0 wt% (a) and 4.0 wt% (b), measured with an applied electric field strength of 0.5 $MVcm^{-1}$. The field strength dependence of the E-A signals monitored at the minimum is shown in (c) for the 3.0 wt% sample and in (d) for 4.0 wt% sample. Absorption spectra are also shown in (a) and (b) by chain lines.

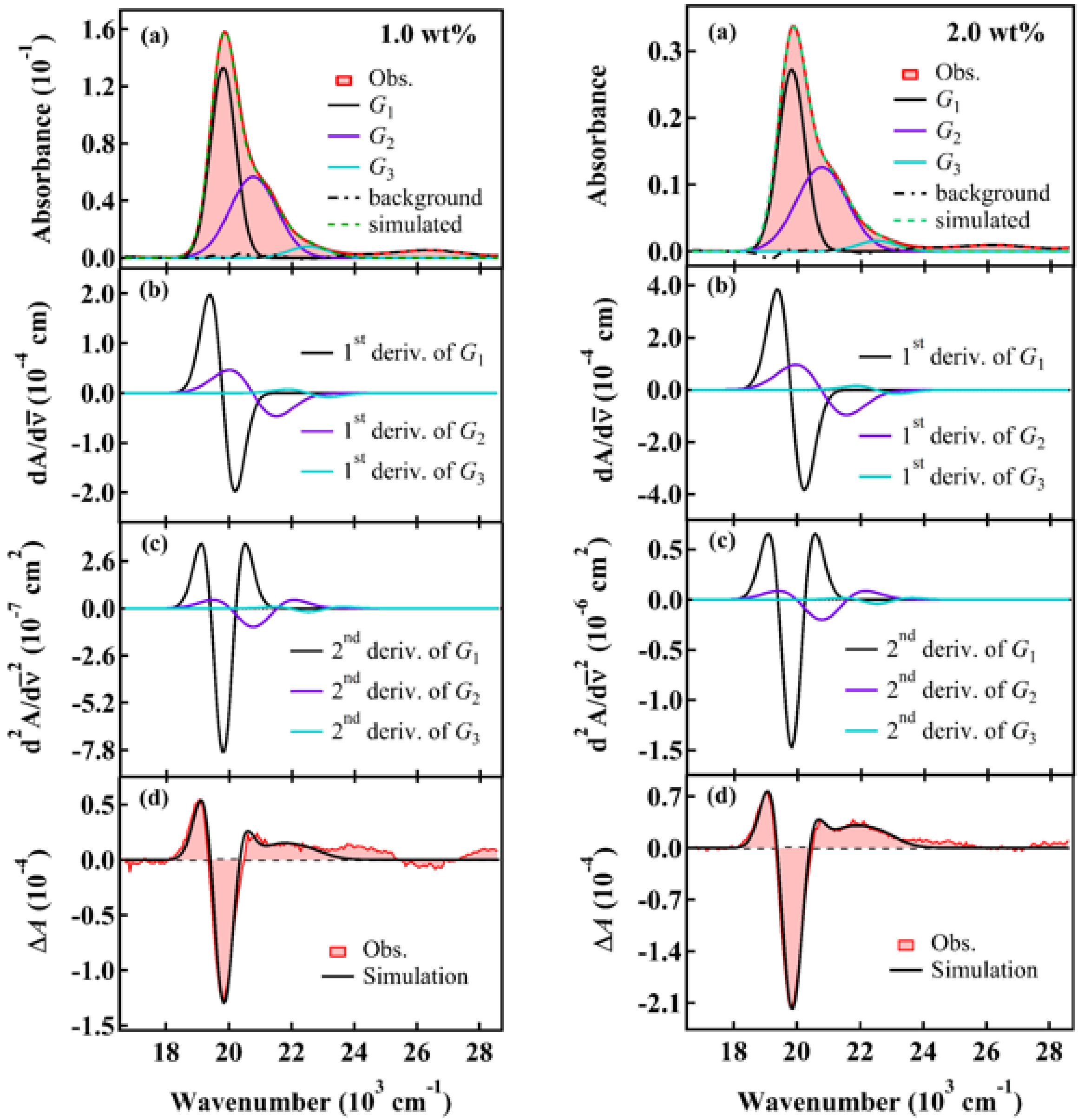

Figure S4. Analysis of the E-A spectra of FL:PVA film with a FL concentration of 1.0 wt% (left) and 2.0 wt% (right). Absorption spectra and their decomposed Gaussian bands (a), the first derivatives of the Gaussian bands (b), the second derivatives of the Gaussian bands (c), and simulated E-A spectra with the observed spectra (d).

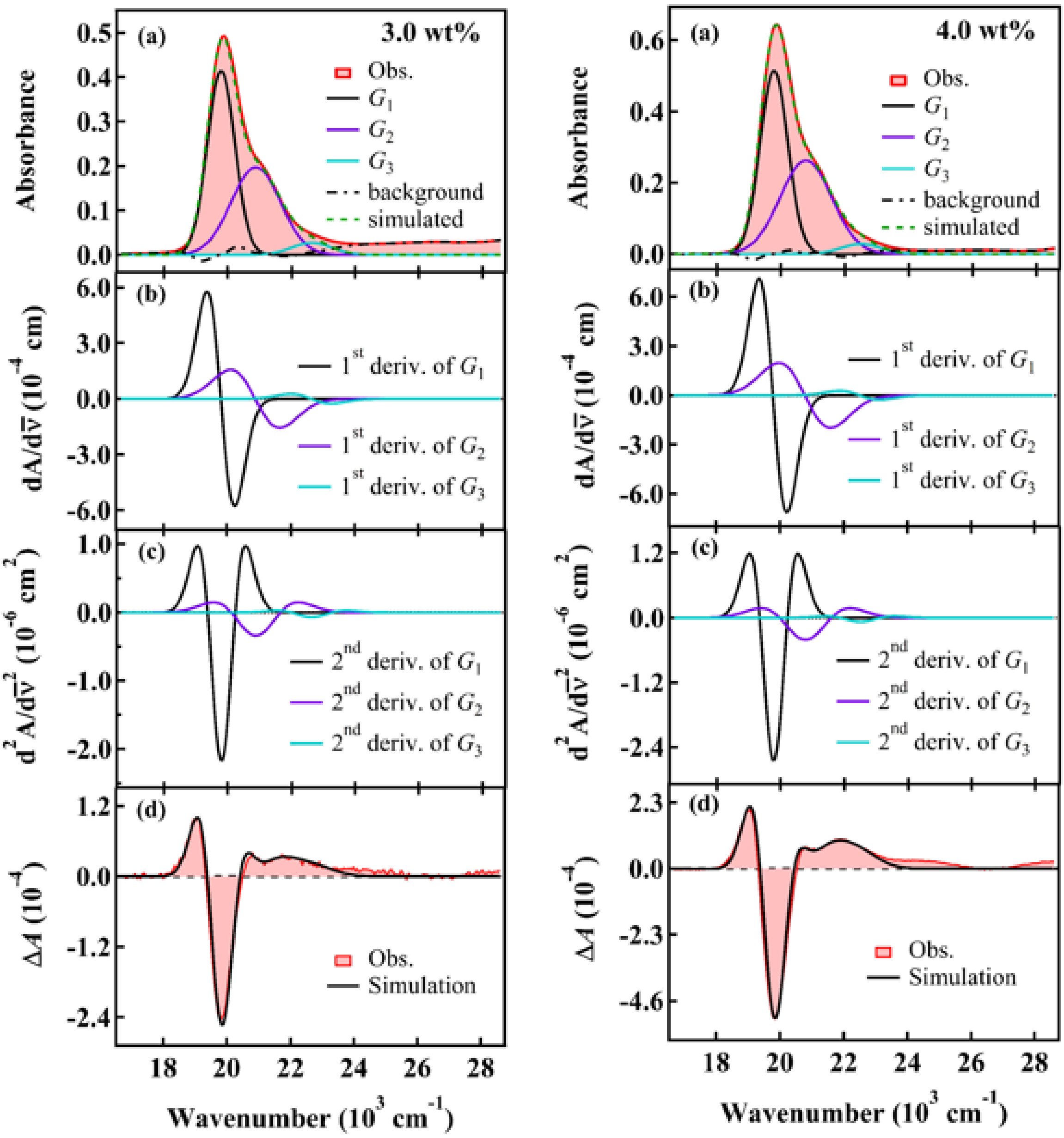

Figure S5. Analysis of the E-A spectra of FL:PVA film with a FL concentration of of 3.0 wt% (left) and 4.0 wt% (right). Absorption spectra and their decomposed Gaussian bands (a), the first derivatives of the Gaussian bands (b), the second derivatives of the Gaussian bands (c), and and simulated E-A spectra with the observed spectra (d).

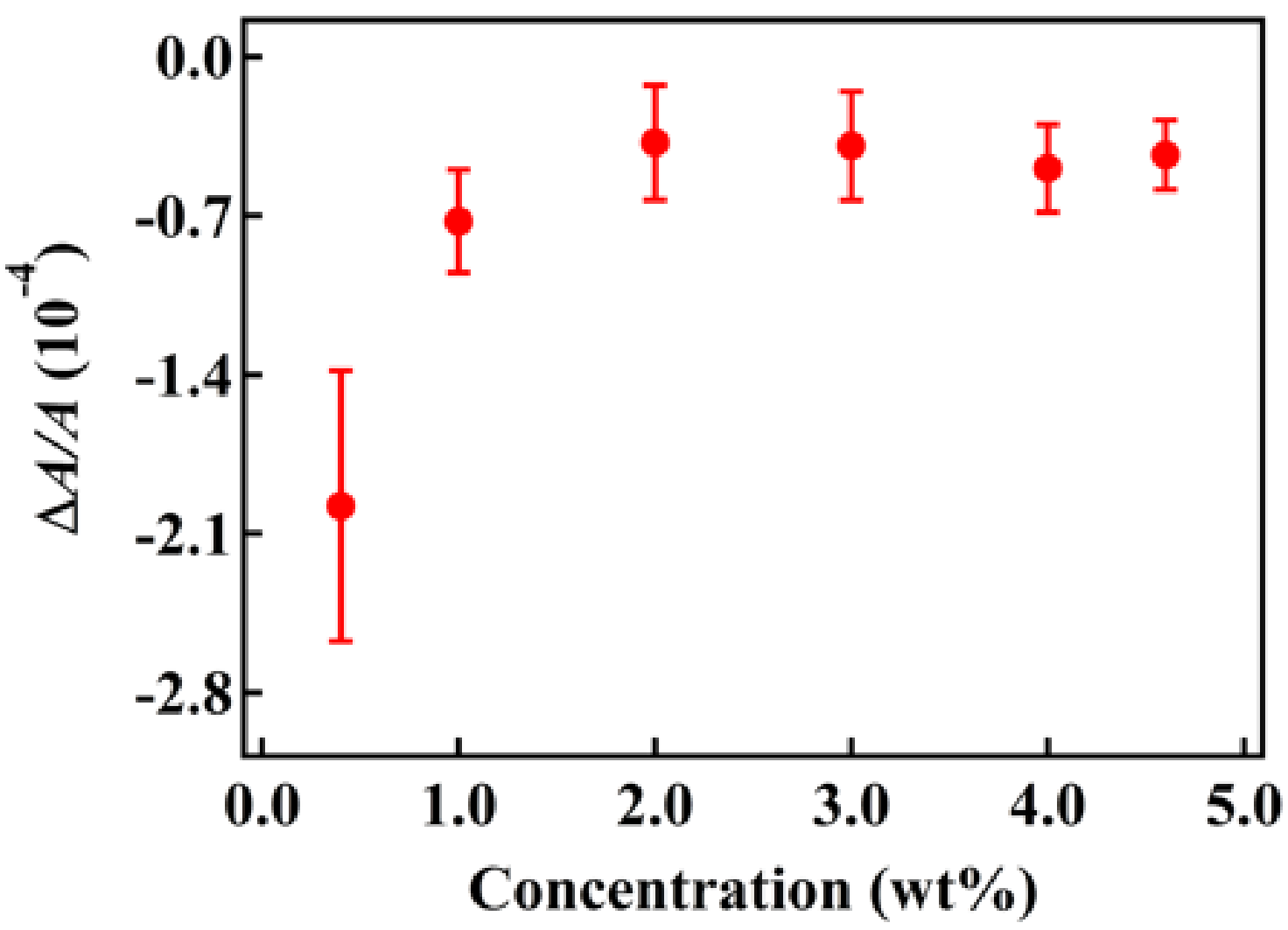

Figure S6, Plots of ΔA/A of FL in PVA as a function of concentration of FL.

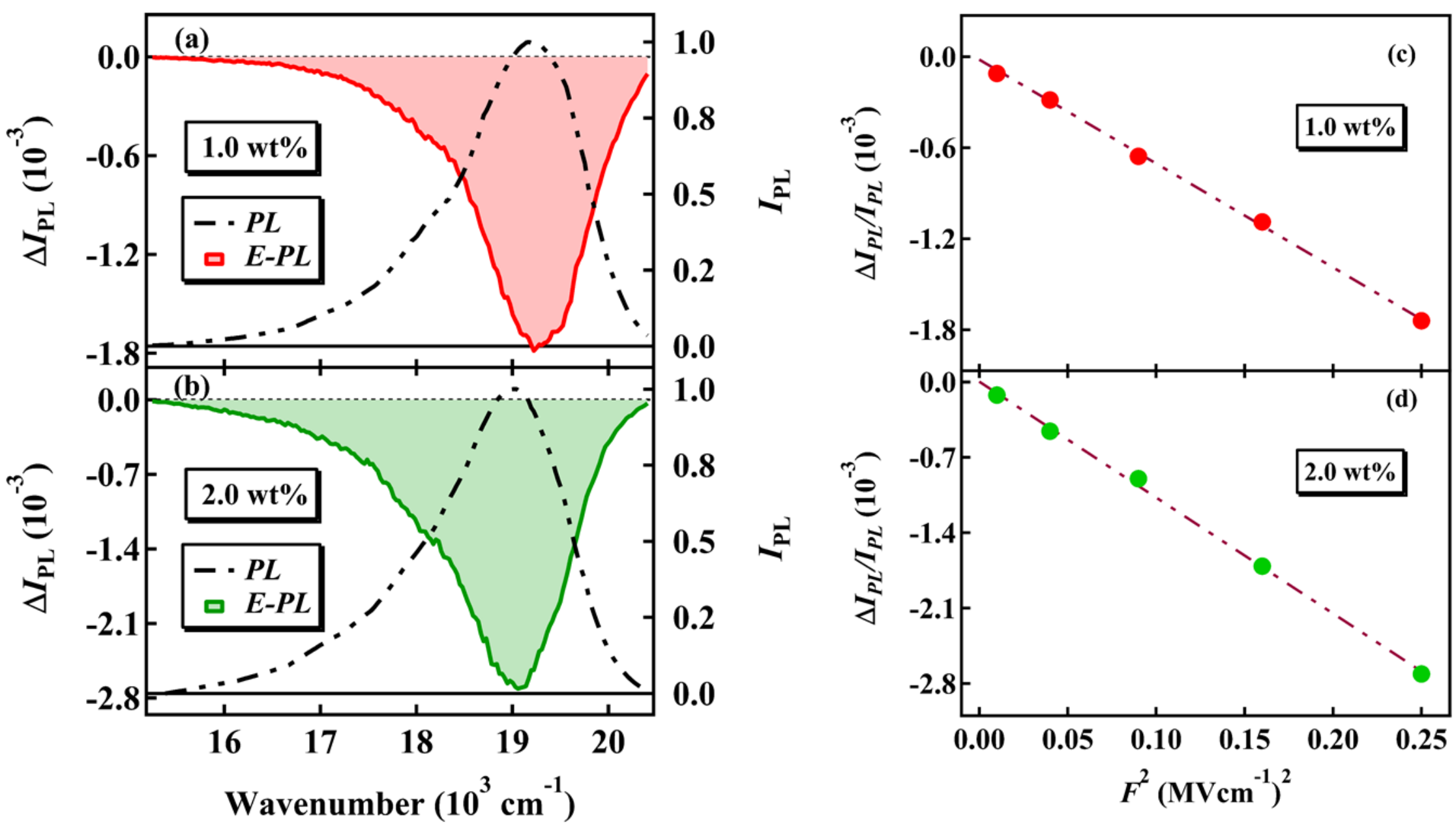

Figure S7. E-PL spectra of FL:PVA film measured with a field strength of 0.5 $MVcm^{-1}$ for the samples with FL concentration of 1.0 wt% (a), 2.0 wt% (b), together with the PL spectra. Applied field strength dependence of the field-induced change in the PL intensity relative to the PL intensity ($\Delta I_{PL}/I_{PL}$) for the samples with FL concentrations of 1.0 wt% (c) and 2.0 wt% (d), monitored at the minimum of the E-PL signal.

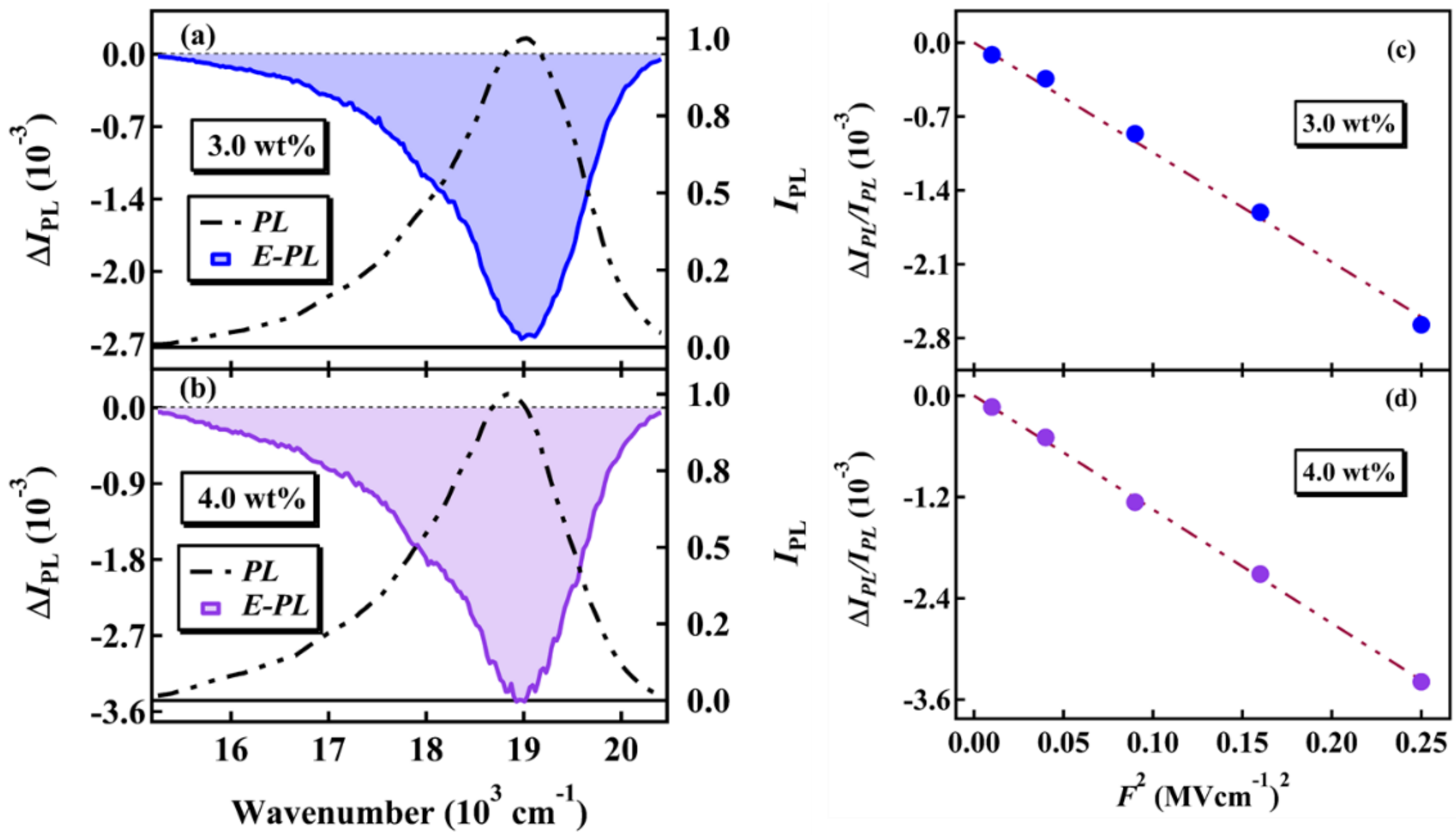

Figure S8. E-PL spectra of FL:PVA film measured with a field strength of 0.5 MVcm^{-1} for the samples with FL concentration of 3.0 wt% (a), 4.0 wt% (b), together with the PL spectra. Applied field strength dependence of the field-induced change in the PL intensity relative to the PL intensity ($\Delta I_{PL}/I_{PL}$) for the samples with FL concentrations of 3.0 wt% (c) and 4.0 wt% (d), monitored at the minimum of the E-PL signal.

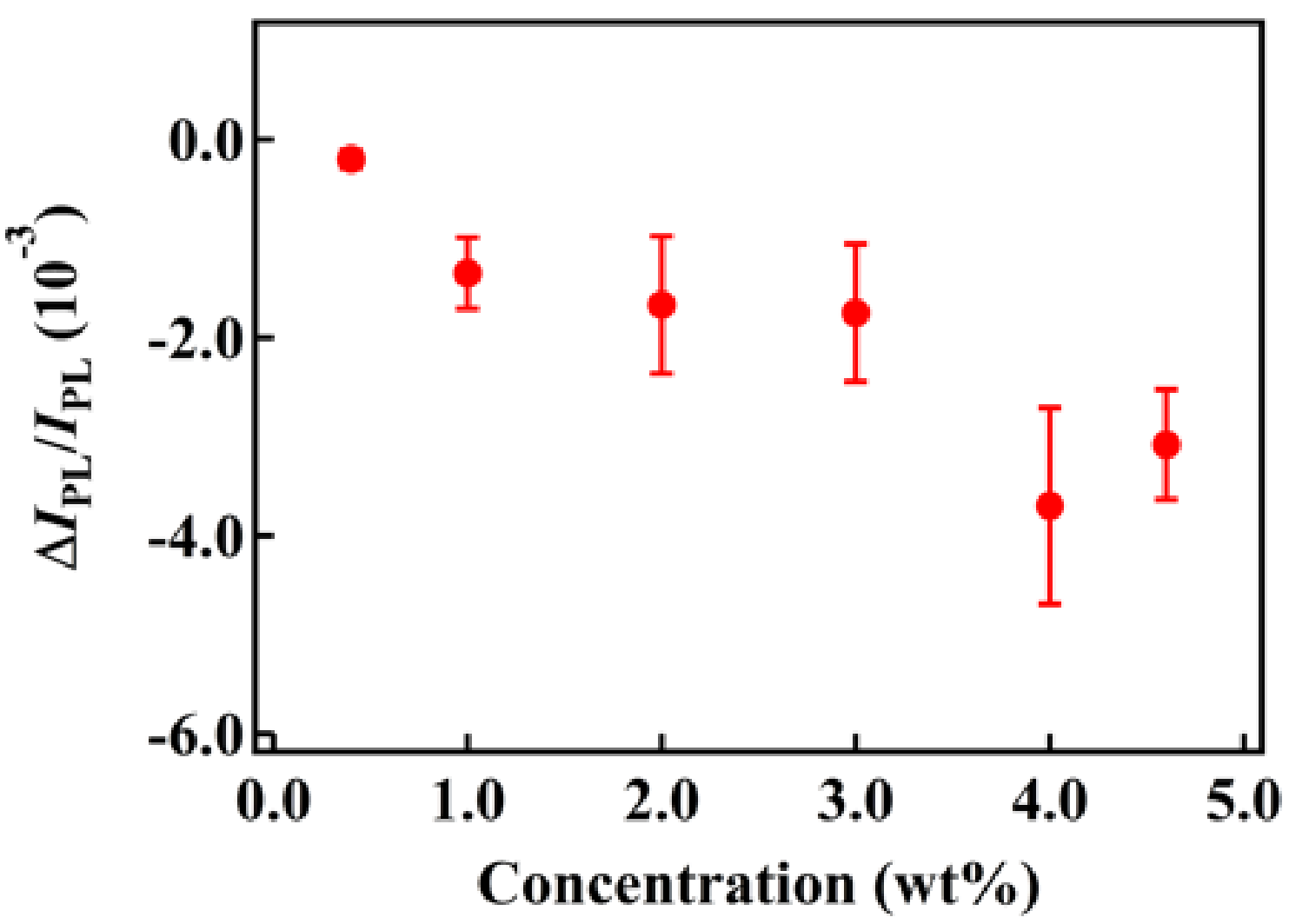

Figure S9. $\Delta I_{PL}/I_{PL}$ of FL:PVA film as a function of FL concentration.

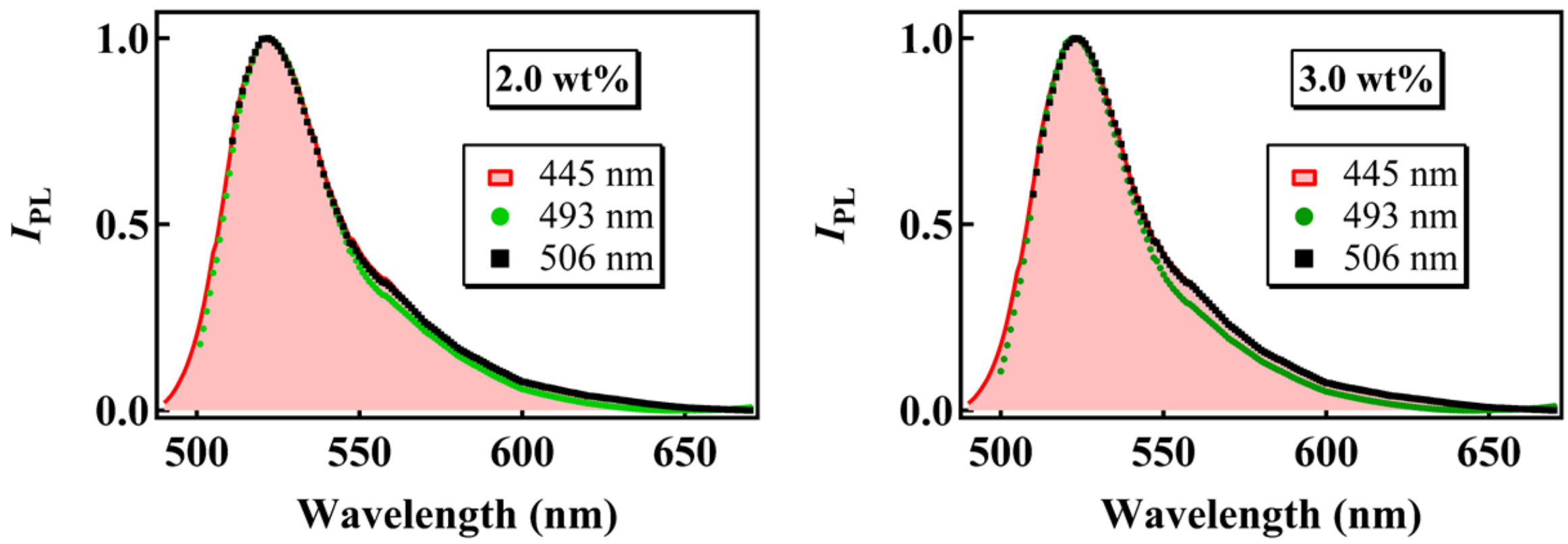

Figure S10. Excitation wavelength dependence of PL spectra of FL:PVA film at different FL concentrations. Excitation wavelengths were 506, 493, 482, 445 nm.

Table S1. The results of the analysis of the E-A spectra of FL in a PVA film at different concentrations.

Concentration (wt%)	Band	Coefficient δ (MV^{-2})	Coefficient θ ($MV^{-2}cm^{-1}$)	$\Delta\bar{\alpha}$ ($Å^3$)	Coefficient η ($MV^{-2}cm^{-2}$)	$\lvert\Delta\boldsymbol{\mu}\rvert$ (D)
0.4	***G*₁**	-0.00021 (± 4 × 10^{-5})	0.05 (± 0.04)	7.1 (± 5.3)	179.0 (± 13.5)	3.9 (± 0.2)
	***G*₂**	-0.00004 (± 4 × 10^{-5})	-0.47 (± 0.17)	-67.6 (± 24.6)	4.3 (± 2.5)	0.6 (± 0.2)
1.0	***G*₁**	-0.00011 (± 3 × 10^{-5})	0.13 (± 0.04)	18.2 (± 5.7)	127.5 (± 24.0)	3.3 (± 0.3)
	***G*₂**	-0.00002 (± 2 × 10^{-5})	-0.25 (± 0.14)	-36.1 (± 19.7)	4.8 (± 2.2)	0.6 (± 0.2)
	***G*₃**	0.00104 (± 0.00024)	0.10 (± 0.03)	13.9 (± 4.8)	6.0 (± 2.7)	0.7 (± 0.2)
2.0	***G*₁**	-0.00005 (± 3 × 10^{-5})	0.06 (±0.05)	8.3 (± 7.2)	83.0 (± 42.2)	2.6 (± 0.7)
	***G*₂**	-0.00004 (± 3 × 10^{-5})	-0.18 (± 0.11)	-25.1 (±16.3)	4.3 (± 1.6)	0.6 (± 0.1)
	***G*₃**	0.00078 (± 0.00043)	0.10 (± 0.04)	13.6 (± 6.4)	6.5 (± 2.1)	0.7 (± 0.1)
3.0	***G*₁**	-0.00005 (± 5 × 10^{-5})	0.07 (± 0.05)	10.4 (± 7.8)	93.3 (± 45.3)	2.8 (± 0.6)
	***G*₂**	-0.00004 (± 1 × 10^{-5})	-0.21 (± 0.08)	-29.6 (±12.0)	5.7 (± 1.7)	0.7 (± 0.1)
	***G*₃**	0.00079 (± 0.00050)	0.03 (± 0.01)	4.2 (± 2.0)	4.9 (± 1.9)	0.6 (± 0.1)
4.0	***G*₁**	-0.00005 (± 3 × 10^{-5})	0.13 (± 0.02)	19.0 (± 3.0)	141.8 (± 15.5)	3.6 (± 0.2)
	***G*₂**	-0.00008 (± 0.00002)	-0.40 (± 0.10)	-52.5 (± 9.5)	6.1 (± 1.8)	0.7 (± 0.1)
	***G*₃**	0.00170 (± 0.00008)	0.03 (± 0.01)	4.0 (± 2.0)	5.3 (± 2.0)	0.7 (± 0.1)
4.6	***G*₁**	-0.00003 (± 3 × 10^{-5})	0.15 (± 0.03)	22.0 (± 4.0)	129.6 (± 12.1)	3.3 (± 0.2)
	***G*₂**	-0.00009 (± 4 × 10^{-5})	-0.24 (± 0.03)	-34.0 (± 4.0)	5.1 (± 2.4)	0.6 (± 0.2)
	***G*₃**	0.00112 (± 0.00050)	0.009 (± 0.001)	1.3 (± 0.2)	11.4 (± 3.0)	1.0 (± 0.1)
